# A High Accuracy Electrographic Seizure Classifier Trained Using Semi-Supervised Labeling Applied to a Large Spectrogram Dataset

**DOI:** 10.3389/fnins.2021.667373

**Published:** 2021-06-28

**Authors:** Wade Barry, Sharanya Arcot Desai, Thomas K. Tcheng, Martha J. Morrell

**Affiliations:** ^1^NeuroPace, Inc., Mountain View, CA, United States; ^2^Department of Neurology, Stanford University, Stanford, CA, United States

**Keywords:** semi-supervised labeling, ECoG labeling, big data, electrographic seizure classifier, epilepsy

## Abstract

The objective of this study was to explore using ECoG spectrogram images for training reliable cross-patient electrographic seizure classifiers, and to characterize the classifiers’ test accuracy as a function of amount of training data. ECoG channels in ∼138,000 time-series ECoG records from 113 patients were converted to RGB spectrogram images. Using an unsupervised spectrogram image clustering technique, manual labeling of 138,000 ECoG records (each with up to 4 ECoG channels) was completed in 320 h, which is an estimated 5 times faster than manual labeling without ECoG clustering. For training supervised classifier models, five random folds of data were created; with each fold containing 72, 18, and 23 patients’ data for model training, validation and testing respectively. Five convolutional neural network (CNN) architectures, including two with residual connections, were trained. Cross-patient classification accuracies and F_1_ scores improved with model complexity, with the shallowest 6-layer model (with ∼1.5 million trainable parameters) producing a class-balanced seizure/non-seizure classification accuracy of 87.9% on ECoG channels and the deepest ResNet50-based model (with ∼23.5 million trainable parameters) producing a classification accuracy of 95.7%. The trained ResNet50-based model additionally had 93.5% agreement in scores with an independent expert labeller. Visual inspection of gradient-based saliency maps confirmed that the models’ classifications were based on relevant portions of the spectrogram images. Further, by repeating training experiments with data from varying number of patients, it was found that ECoG spectrogram images from just 10 patients were sufficient to train ResNet50-based models with 88% cross-patient accuracy, while at least 30 patients’ data was required to produce cross-patient classification accuracies of >90%.

## Introduction

One of the major challenges in epilepsy treatment is the ability to reliably assess patient outcomes (Engel, 2011; Engel et al., 2013). Patient reports of seizures can be unreliable and incomplete because patients may be amnestic for seizures, may not document their seizures, or because seizures often occur during sleep (Bazil et al., 2004; Kerling et al., 2006; Hoppe et al., 2007). A potential solution to this problem is to automatically detect and count electrographic seizures from electrocorticographic/physiological data captured using devices such as implanted neuromodulation devices (Ryapolova-Webb et al., 2014; Skarpaas et al., 2019), or health-monitoring wearables that record long-term ambulatory patient data (Bruno et al., 2018; Regalia et al., 2019).

Machine and deep learning models previously developed for detecting electrographic seizures from physiological data have demonstrated excellent performance (Thodoroff et al., 2016; Acharya et al., 2018; O’Shea et al., 2018; Ansari et al., 2019; Roy et al., 2019). However, most of these models are either patient-specific or trained on small subsets of patients, which limits their applicability to new patients or patients in whom only small datasets are available. Additionally, previous models were mostly trained on electroencephalographic (EEG) data captured during intracranial EEG diagnostic monitoring, which may vary substantially from data captured in a long-term ambulatory setting and hence may not translate effectively to data captured outside the clinical setting (Ung et al., 2017; Baumgartner and Koren, 2018; Sun et al., 2018).

Large multi-patient ambulatory electrocorticographic (ECoG) datasets obtained during clinical trials of the NeuroPace^®^ RNS^®^ System may facilitate the development of machine learning (ML) algorithms for seizure detection (Morrell, 2011; Bergey et al., 2015; Skarpaas et al., 2019). However, training of a supervised machine/deep learning electrographic seizure classifier (ESC) requires ECoG datasets that contain training labels to mark both electrographic seizure and non-seizure portions in the datasets. In computer vision classification tasks involving classifying everyday objects such as cats and dogs, the task of labeling large datasets is often crowdsourced to non-specialist workers around the world (example: Amazon Mechanical Turk^1^, FigureEight^2^, Samasource^3^). A similar crowdsourcing technique may not be suitable for labeling ECoG datasets given their complex nature. Individuals specifically trained on labeling ECoG records are needed, and even then, interlabeler agreement is not guaranteed (Halford et al., 2011; Halford et al., 2015). The process of creating training labels for large ECoG datasets can thus be laborious and in many cases may even deter the development of powerful ML models.

Electrographic seizure patterns are often stereotypical within individual patients in time windows spanning a few months to many years (Manford et al., 1996). Hence, if a representative electrographic seizure pattern in a patient is manually labeled, a method to automatically pre-label other similar electrographic seizure patterns in the patient would substantially expedite the process of manual review and labeling of ECoG data. Further, if this method is generic and can be readily applied to individual patient’s ECoGs without the need for patient-level customizations, the solution could easily scale to large multi-patient datasets. ‘One-shot’ and ‘few-shot’ learning techniques for classifying objects with just one or a few training examples of each class are currently being explored in computer vision with promising results (Vinyals et al., 2016; Snell et al., 2017). This paper describes a semi-supervised labeling technique, based on unsupervised features extracted by a pre-trained deep convolution neural network, for expediting the process of labeling the large multi-patient ECoG dataset captured with the RNS System.

Electrographic seizure patterns can vary substantially between patients due to differences in seizure onset zones, disease etiologies, and location and orientation of recording electrodes (Haas et al., 2007). Given the heterogeneous nature of electrographic seizures, development of ESCs that generalize in new patients can be a complex problem, one on which the rules- or feature-based algorithms may fare poorly. Deep learning models can learn rules and features directly from data and are particularly well-suited for problems that are too complicated to craft rules (LeCun et al., 2015). Many previous papers have demostrated the superiority of deep learning based EEG classification models over traditional machine learning models (Arora et al., 2018; Zeng et al., 2018). In this paper several different deep learning models of varying architectures and depths have been trained to classify ECoG records as electrographic seizures or non-seizures.

Performance of deep learning models generally improves with depth. However, the majority of the previous work on training deep learning models for seizure detection have used convolutional neural networks (CNNs) that are <10 layers deep (Roy et al., 2019),which is relatively shallow compared to the more recently developed contest-winning models (Russakovsky et al., 2015; He et al., 2016). This is presumably due to the limited amount of EEG training data available in the previous studies. In the current study, training and validation experiments were performed on the large multi-patient ambulatory ECoG dataset (137,985 ECoG records from 113 patients) captured with the RNS System. Because of this abundance of data, the deeper 18-layer ResNet18 and 50-layer ResNet50 (He et al., 2016) architectures were experimented with, in addition to shallower CNNs. ResNet models contain residual blocks which have been shown to alleviate the problem of exploding and vanishing gradients which can arise in deep neural network architectures and were chosen for this reason (He et al., 2016).

Despite the tremendous progress made by CNNs in classifiying 2 dimensional (2D) image data (LeCun et al., 2015), most of the previous work in training CNNs for ECoG/EEG classification has focused on using raw time-series signals as input to one dimensional CNNs (Ullah et al., 2018; Roy et al., 2019; Yildirim et al., 2020). Very few studies have explored converting ECoG time-series signals to spectrogram images for training 2 dimensional CNNs, and even the ones which do have demostrated their methods on relatively small EEG datasets (Kuanar et al., 2018; Vrbancic and Podgorelec, 2018). Validating 2D CNN training on large multi-patient ECoG spectrogram image datasets will certainly add confidence in this technique, and will encourage similar EEG/ECoG classification studies to leverage the latest developments in 2D CNN image processing, potentially accelerating the rate of neuroscience discoveries.

Although it is widely accepted that large ECoG training datasets will lead to better model generalizability to new patients (LeCun et al., 2015), to the best of our knowledge, characterizing electrographic seizure classification accuracy in held-out patients as a function of the number of patients whose data was used for training, has not been performed. In this paper, CNN models were trained with ECoG records from 10 to 80 patients, in increments of 10, to determine the generalizability of models trained with ECoG records from varying numbers of patients. All trained models were tested on expert-labeled ECoG records from 80 additional held-out patients (i.e., in addition to the 113 patients mentioned above) not used for training or validation.

The work in this paper significantly adds to the existing body of literature on labeling and training electrographic seizure classifiers in several ways. First, it introduces a semi-supervised labeling method for rapid manual-labeling of large ECoG datasets. Second, it validates the use of ECoG spectrogram images as inputs for training convolutional neural networks by producing trained classification models with very high (>95%) cross-patient classification accuracies. Third, it establishes a new benchmark cross-patient electrographic seizure classification accuracy level for ambulatory ECoG records. Fourth, it characterizes classification accuracy as a function of the amount of training data, thereby guiding the neuroscience community on data collection requirements for solving similar ECoG/EEG classification problems.

## Methods

The present study’s dataset comes from clinical trials of 256 patients treated with the NeuroPace^®^ RNS^®^ System (Bergey et al., 2015). One hundred and ninety three randomly selected patients were used for the analyses in this study.

All study protocols were approved by the US FDA and the institutional review boards of the participating investigation sites. All participants gave written informed consent. The RNS System Feasibility, Pivotal and LTT studies are registered on clinicaltrials.gov (NCT00079781, NCT00264810, and NCT00572195).

### The RNS System

**FIGURE 1:**
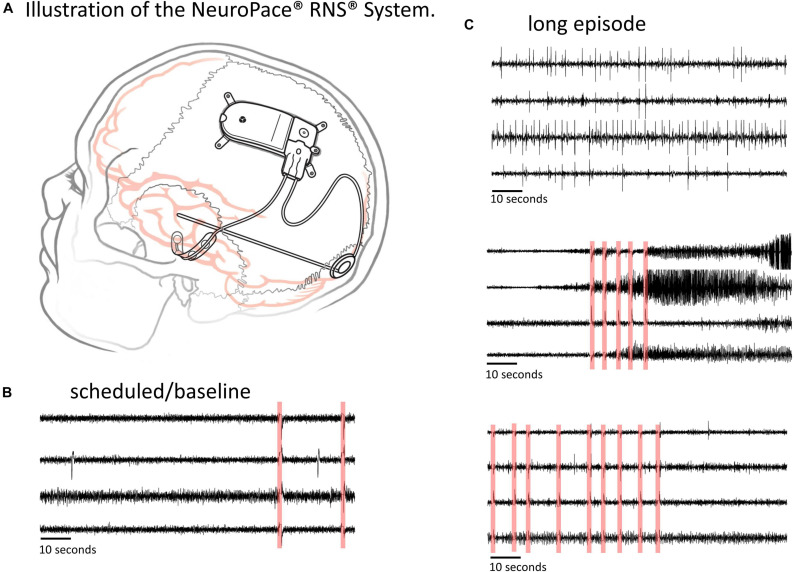
The RNS System and types of ECoG data captured. **(A)** Illustration of the NeuroPace RNS System. The neurostimulator is placed in the skull and connected to up to two leads, each of which contains four electrode contacts. The leads can be cortical strip leads, depth leads or a combination. **(B,C)** Examples of 4-channel electrocorticographic (ECoG) records for a single patient captured with the RNS System. **(B)** Scheduled/baseline ECoG record. **(C)** ‘Long Episode’ (LE) ECoG records (i.e., ECoG records with detection of long abnormal patient-specific patterns). LE ECoG records can contain a variety of abnormal ECoG activity on one to several ECoG channels. Stimulation artifacts are highlighted in red in panels **(B,C)**. Stimulation artifact consists of short blanks in data followed by a brief amplifier recovery artifact.

The NeuroPace^®^ RNS^®^ System is an FDA approved adjunctive treatment for patients with medically intractable partial onset epilepsy having 1-2 seizure foci. Details about the RNS System and the types of data it captures can be found in several previous publications (Morrell, 2011; Bergey et al., 2015; Desai et al., 2019b; Skarpaas et al., 2019). Briefly, the RNS System (Figure 1A) consists of a closed-loop responsive neurostimulator device that is placed in the skull. One or two quadripolar depth or strip leads are connected to the device and implanted at the seizure foci. The RNS System continuously senses brain activity and sends electrical stimulation when patient-specific abnormal patterns, as defined by the physician, are detected.

### ECoG Acquisition and Patient Selection

ECoG records captured with the RNS System have a sampling rate of 250 Hz per channel and are typically 90 s in duration, however length may vary to a maximum of 180 s. An ECoG record typically contains four channels of ECoG activity. A variety of recording triggers save ECoG records including, time(s) of day (scheduled ECoG records), detection of long abnormal patient-specific patterns (long episode ECoG records), and ECoG activity that saturates the recording amplifiers (saturation ECoG records). About half of all ECoG records captured with the RNS System are long episode (LE) ECoG records. LE ECoG records can contain varying degrees of abnormal epileptiform events on one or more ECoG channels and are the only type included in this study. Figures 1B,C shows four example ECoG records captured in one RNS System patient.

In the remainder of this paper, the term ‘ECoG record’ refers to an ECoG data file with up to 4 channels of ECoG data, and the term ‘ECoG channel’ refers to each channel of ECoG activity within an ECoG record. The terms ‘electrographic seizures,’ ‘seizures,’ and ‘sz’ are used interchangeably to refer to electrographic seizures; and ‘electrographic non-seizures,’ ‘non-seizures,’ and ‘nsz’ are used to refer to electrographic non-seizures.

**FIGURE 2:**
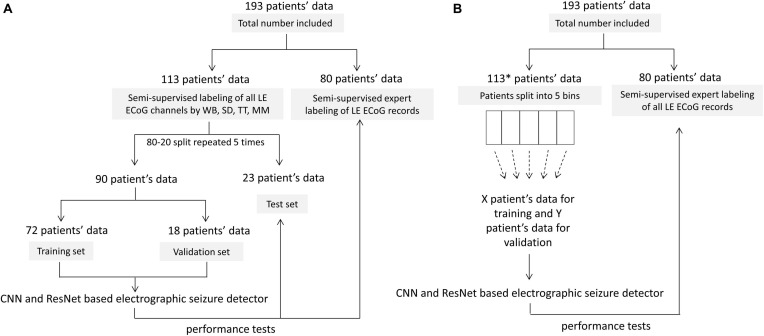
Patient splits for labeling, training, validating and testing electrographic seizure classifier models. **(A)** Patient splits for training and testing electrocorticographic (ECoG) channel-level and ECoG record-level classification models with 80% patients’ data used for training and validation, and 20% patients’ data used for testing. The trained models were tested on individual ECoG channels within ECoG records from the 23 held-out patients in each training fold, to generate ECoG channel-level classification test metrics. The trained models were additionally tested on an independent expert-labeled dataset from 80 patients with seizure or non-seizure ECoG record-level labels. In this case, model predictions on individual ECoG channels within ECoG records were combined with the OR operator applied to the seizure classification to produce ECoG record-level predictions. These were compared with expert labels to generate ECoG record-level classification test metrics. **(B)** 10-80 patient splits for characterizing model performance as a function of amount of training data. *8 out of 113 patients did not have ECoG channels with the ‘seizure’ label and were not included in this analysis. This was done to avoid creating training datasets (especially the ones with data from small number of patients) with highly skewed number of examples of seizure and non-seizure classifications. The remaining 105 patients were split into 5 bins (with 21 patients in each bin) based on the number of labeled ECoG records available in the patient. Patients were uniformly and randomly selected from each of the 5 bins to create the 10-80 patient training sets. The 20 patient training set, for example, contained four patients randomly selected from each of the 5 bins. The trained models were tested on ECoG records from 80 patients independently labeled by an expert. Note that unlike in panel **(A)**, the only test dataset used in this case was the expert labeled dataset from 80 patients.

**FIGURE 3:**
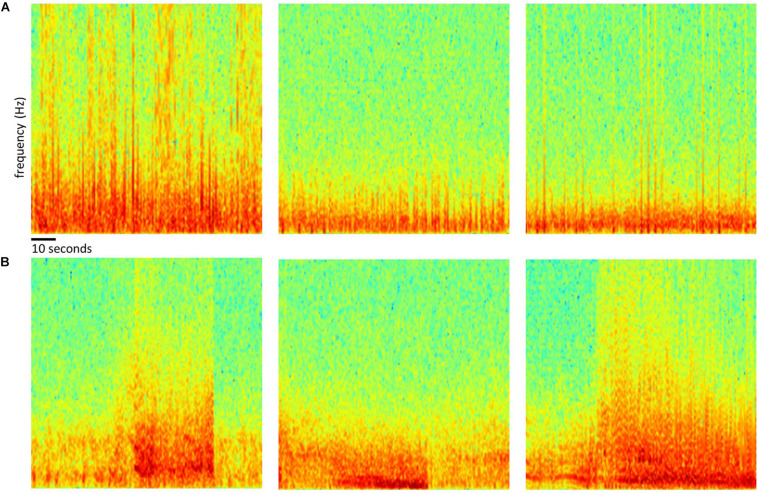
Examples of spectrogram images of ECoG channels. Top row **(A)** shows examples of spectrogram images computed from electrocorticographic (ECoG) channels manually labeled as ‘electrographic non-seizure.’ Bottom row **(B)** shows examples of spectrogram images computed from ECoG channels labeled as ‘electrographic seizure.’

Of the 256 patients enrolled in the RNS System clinical trials, 193 were randomly selected for inclusion in this study. Data from all 256 patients could not be processed due to limited human labeler time resources. Data from 113 patients were used to train, test and validate the ESC, and data from the remaining 80 patients were used only for testing by comparison of the ESC’s classification scores with those of a board certified epileptologist. Figure 2 outlines the data split from the 193 patients, and Figure 3 shows example spectral image of ECoG channels labeled as non-seizures (top) and seizures by a human labeler.

### Patient-Specific 2D Embedding and Clustering of ECoG Records

**FIGURE 4:**
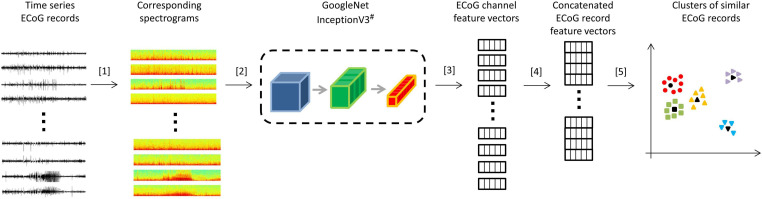
Steps for creating 2D embeddings of patient-specific ECoG records. [1] Each channel in the time series electrocorticographic (ECoG) record was converted to a spectrogram image after removal of stimulation artifacts. [2] Spectrogram image (size 224 × 224 × 3) of each ECoG channel was passed to a pre-trained GoogLeNet Inception-V3 model for feature extraction. [3] The convolutional neural networks (CNN) extracted a feature matrix of dimensions 8 × 8 × 2048 for each spectrogram image. The feature matrix was flattened to create a feature vector of length 131,072. [4] Four feature vectors corresponding to the four channels in an ECoG record were concatenated to create a feature vector of length 524,288 for each ECoG record. Vectors of zeros were substituted for missing channels. [5] Dimensionality reduction with PCA and t-SNE was performed on the concatenated feature vectors to represent ECoG records in 2-dimensional patient-specific embedding spaces. Unsupervised clustering of the resulting 2D data was performed. ECoG records closest to cluster centers (shown in black) were identified and presented to the human labeler.^#^ Cartoon representation of the GoogleNet Inception-V3 CNN model. Please refer to https://cloud.google.com/tpu/docs/inception-v3-advanced for details about the GoogleNet Inception-V3 model.

All analyses were performed using Python 3.5. Patient-specific 2D embeddings of all LE ECoG records from 193 patients were created using unsupervised feature extraction and dimensionality reduction techniques. Data preparation involved removing any stimulation artifacts present in the ECoG records (Desai et al., 2019a). In brief, blanked portions of ECoGs (Figures 1B,C) were identified and marked along with 10 samples preceding and 30 samples following the detected artifact. ECoG data flanking either side of the marked ECoG data were concatenated to delete stimulation and any amplifier recovery artifact. Spectrograms of the time-series ECoG data were computed using Tensorflow’s built-in function tensorflow.contrib.signal.stft. Since high frequency (>90 Hz) seizure and interictal activity is often observed in ECoG data captured with the RNS System, frequencies from 0 Hz to the Nyquist frequency (i.e., 125 Hz; sampling rate = 250 Hz) were included in the spectrograms. The resulting grayscale spectrograms were resized to (299 × 299) using Tensorflow’s built-in function tensorflow.image.resize_nearest_neighbor, and expanded to 3 identical color channels using Tensorflow’s built-in function tensorflow.image.grayscale_to_rgb (Figure 4 step [1]). Expansion of spectrograms to 3 channels was performed because the pre-trained CNN (GoogLeNet Inception-V3) used for feature extraction requires the input data to have shape (299 × 299 × 3). The resulting 3 color channel spectrograms were passed through the pre-trained GoogLeNet Inception-V3 model for feature extraction (Figure 4 step [2]). Tensorflow code for converting the time-series ECoG data to spectrograms and extracting features using the pre-trained GoogLeNet Inception-V3 model is provided in the Supplementary Material. A similar technique for embedding time-series ECoG data in 2 dimenional surfaces for differentiating ECoGs by patient outcomes was previously published by NeuroPace and are described in Desai et al. (2019a).

The extracted features (dimensions 8 × 8 × 2048) were flattened resulting in a vector of 131,072 floating point numbers for each channel of ECoG data (Figure 4 step [3]). Features vectors from the 4 channels in ECoG records were concatenated to produce ‘ECoG-record-vectors’ that had 524,288 features. If less than 4 channels were present in ECoG records (which happened in a small fraction of ECoG records), zero-filled vectors were used in the missing channel’s place. Principal Component Analysis (PCA) was applied to randomly selected 1,024 ECoG-record-vectors to derive a mapping function between the 524,288 features and the top 50 principal components. The mapping function was then applied to all ECoG-record-vectors in a patient to produce a reduced feature vector with 50 principal components for each ECoG record. The resulting 50 components were then passed to the t-SNE (t-distributed stochastic gradient descent) algorithm to represent all ECoGs in a patient-specific further reduced 2-dimensional embedding space.

Bayesian Gaussian Mixture (BGM; python function: sklearn.mixture.BayesianGaussianMixture) was used for automatically clustering ECoG records represented in the patient-specific 2D embedding spaces. The BGM clustering technique was chosen over other popular clustering methods such as k-means, spectral, and dbscan (density based spatial clustering) because of its ability to infer the number of clusters from the data and because it produced more sensible cluster identifications compared to the other clustering methods that were evaluated. The sklearn.mixture.BayesianGaussianMixture function takes as input the maximum number of components/clusters (n_components). Depending on the data, the BGM model can choose not to use all the components. Therefore, the number of effective components/clusters can be smaller than the number specified in n_components. For clustering ECoG records in each patient using BGM, the n_components attribute was set as maximum of [number of ECoG records in patient/100] and 10. For example, for a patient with 5,000 ECoGs, n_components would be 50, and for a patient with 200 ECoGs, n_components would be 10.

Similar to observations in Macosko et al. (2015) where authors clustered gene expression data, preliminary studies by NeuroPace also showed superior clustering in the 2 dimensional t-SNE output dataset compared to clustering in the original high dimensional dataset. This is presumably because of a frequently observed phenomenon called ‘curse of dimensionality’ in which clustering algorithms lose effectiveness in high dimensional spaces (Verleysen and François, 2005).

### Labeling of ECoG Records

All LE ECoG records in 193 patients were manually labeled and verified using a 2-step method. In 113 out of 193 patients, ECoG records were given one of six labels: ‘ictal,’ ‘interictal,’ ‘baseline,’ ‘noise,’ ‘low voltage fast only,’ or ‘unsure,’ and channels in the ECoG record with the designated activity were selected by author WB. WB was specifically trained in labeling activity in ECoG records captured with the NeuroPace RNS System over a 2 month period by authors SD, TT and MM. Both time-series waveforms and spectrogram views of data were used to guide ECoG labeling. In case of labeling ambiguity, multiple reviewers (authors SA, TT, and MM) provided inputs to ensure accurate labeling. The ‘ictal’ label was selected if there was clear evolution of baseline activity into electrographic seizure that lasted at least 10 s. The ‘unsure’ label was only selected when reviewers could not reach a consensus on the activity type and ECoG records with the ‘unsure’ label were not used for training, validation or testing. ECoG channels with ‘interictal,’ ‘baseline,’ ‘noise,’ and ‘low voltage fast only’ labels formed the ‘electrographic non-seizure’ classification while ECoG channels with the ‘ictal’ label were used as the ‘electrographic seizure’ classification. When cluster centroid ECoG records were manually labeled, the remaining ECoG records within the cluster were automatically pre-assigned the same label.

The next step involved manual verification of the pre-assigned labels. Thumbnails of ECoG records within each pre-labeled cluster were displayed for label verification in a sorted order based on their Euclidean distance to the centroid. If a pre-assigned channel label did not match the activity observed on that ECoG channel, the label was manually corrected by author WB after consulting with additional reviewers as necessary. Thumbnails of 15 ECoG records were displayed on each page for label verification. Therefore if an ECoG cluster contained 90 ECoG records, 6 pages of ECoG records were displayed with page 1 containing ECoG records that were closest to the centroid and likely requiring no corrections to pre-assigned labels. Whereas page 6 contained ECoG records that were farthest from the centroids and hence was more likely to contain ECoG records that required pre-assigned label corrections compared to previous pages. When a closer look at activity in a ECoG was desired, the thumbnail could be expanded to display high resolution versions of the ECoG records in time series and spectrogram view.

To compare the performance of the trained models with those of an independent ECoG rater, an additional labeled test ECoG dataset was created. In the remaining 80 patients, a board certified epileptologist independently labeled centroid ECoG records as either ‘electrographic seizure’ or ‘electrographic non-seizure.’ A total of 262 and 333 ECoG records were labeled as either seizure and non-seizure, respectively, by the epileptologist. Additionally, the expert only provided labels at the ECoG record level, without identifying the channels in the ECoG record to which the label applied. That is, the seizure label was assigned to an ECoG record if any of the channels in the ECoG record contained an electrographic seizure, and the non-seizure label was assigned otherwise. To compute labeler agreement percentage, author WB additionally labeled all 595 expert-labeled ECoG records.

### ECoG Preprocessing for Training CNN

ECoG records that were less than 80 s and greater than 100 s were repeated or cropped to 90 s in length respectively. ECoG records shorter than 80 s had a portion equal to the disparity duplicated from the beginning of the record and concatenated onto the start. Experiments were repeated with zero padding applied to short ECoG records. In this case, instead of concatenating duplicate portions of the ECoG, a vector of zeros was concatenated onto the start to create the 90 s ECoG. ECoG records greater than 100 s had 60 s before and 30 s after the storage trigger (detection of LE) selected, the remaining portions of the ECoG record were discarded.

Stimulation artifact rejection was performed on all ECoG records as described in section “Patient-Specific 2D Embedding and Clustering of ECoG Records.” Model training and testing experiments were repeated without stimulation artifact rejection to assess the impact of this step on the model’s classification performance.

To facilitate additional ESC model training and testing, spectrogram images of ECoGs were saved in folders organized by class labels and patient IDs. This made it convenient to perform error analyses for the different model architecture and hyperparameter selections. Further, this type of data organization made it stratightforward to apply several of Keras’s built-in functions (such as ImageDataGenerator.flow_from_directory function) for reading large datasets in batches for model training and testing. The code used for converting ECoG data to spectral images and saving them in.PNG format is provided in the Supplementary Material section. Briefly, Matplotlib’s built-in function matplotlib.pyplot.specgram with window size 256 and step size 128 was used for creating the spectrograms (spanning 0-125 Hz on the frequency axis), and saved as RBG images using the ‘jet’ colorsmap. Pixel values in the RGB images were scaled between −1 and + 1 which is a standard preprocessing step for training CNN models. ECoG classification experiments were repeated with spectrograms images saved using the ‘grayscale’ colormap in which case spectrogram images were saved with the 3 color channels having the same value.

### Model Training, Validation, and Testing

#### Experiments to Test the ECoG-Channel Level and ECoG-Record Level Classification Performance of Trained ESC Models

The 113 patients with ECoG channel level labeling were randomly divided into three groups: 72 patients for training, 18 patients for validation and 23 patients for testing. This was repeated five times for creating 5 folds of data for training, validation and testing (Figure 2A). In the training dataset, the majority classification (non-seizure class in all training folds) was randomly downsampled to match the number of training examples in the seizure and non-seizure classes. CNN models were trained to classifiy each ECoG channel (note that each ECoG record can contain up to 4 ECoG channels, see section “ECoG Acquisition and Patient Selection” for details) as electrographic seizure or non-seizure. The trained models in each of the 5 folds were tested on ECoG channels in the 23 patients held-out in that fold.

Model performance was also tested on ECoG records from 80 patients independently labeled by an epileptologist. Model predictions for ECoG records were derived by applying the OR operator to the seizure classification. Consequently, if any of the ECoG channels in the ECoG record were predicted as a seizure by the trained model, the ECoG record was labeled a seizure. A non-seizure label was applied only if all ECoG channels in the ECoG record were predicted as non-seizures by the trained model. Table 1A shows the number of patients, ECoG records and ECoG channels in each of 5 folds.

**TABLE 1A:** Number of patients, seizure and non-seizure ECoG channels in the training, validation and test datasets in each fold (and average across the 5 folds) for testing ECoG channel-level and ECoG record-level classification performances.

**Fold**	**Training**			**Validation**			**ECoG channel-level testing**			**ECoG record-level testing**		
	**# of pts**	**# of SZ ECoG channels**	#of NSZ ECoG channels*	**# of pts**	**# of SZ ECoG channels**	**# of NSZ ECoG channels**	**# of pts**	**# of SZ ECoG channels**	**# of NSZ ECoG channels**	**# of pts**	**# of SZ ECoG records**	**# of NSZ ECoG records**
1	72	81,573	188,858	18	21,692	40,239	23	36,918	42,477	80	262	333
2	72	78,218	174,949	18	33,398	35,751	23	28,567	60,874	80	262	333
3	72	103,572	172,136	18	8,381	36,832	23	28,230	62,606	80	262	333
4	72	91,214	150,498	18	24,957	56,364	23	24,012	64,712	80	262	333
5	72	100,983	205,036	18	15,917	25,066	23	23,283	41,472	80	262	333
**Avg**	**72**	**91,112**	**178,295**	**18**	**20,869**	**38,850**	**23**	**28,202**	**54,428**	**80**	262	333

*ECoG = electrocorticographic; NSZ = non-seizure; pts = patients; SZ = seizure. ^∗^Shown here is the total number of non-seizure ECoG channels available in the training dataset. Note that the non-seizure ECoG channels were randomly downsampled to match the number of seizure ECoG channels to create balanced 50/50 class splits for training. Bold values are average of 5 folds.*

#### Gradient-Based Saliency Maps

To gain some understanding of features learned by the trained CNNs, and to ensure that classification is based on relevant portions of the spectrograms, saliency maps (Simonyan et al., 2013) of the trained classification model were created using the built-in keras API, vizualize_saliency. Saliency maps are computed as the gradient of the output with respect to the input, and highlight the input regions in the datasets that contribute most toward the output classification.

#### Trained Model’s Generalizability to Other Epilepsy Datasets of Time-Series Brain Recordings

To test the trained ESC models’ generalizability to EEG datasets captured with devices other than the RNS System, the models were evaluated on the TUH EEG Seizure Corpus (v.5.1.0)^4^ (Shah et al., 2018), the largest publicly available EEG dataset. Therein, 2,915 annoted seizures had an onset at >45 s into the EDF data files and were used for testing. 90 s of EEG data spanning 45 s respectively on either side of annotated seizure start times were converted to RGB spectrogram images using the methods described in section “ECoG Preprocessing for Training CNN.” The only differences in the data processing steps were (1) skipping the stimulation artifact rejection step since it is irrelevant to EEG data and (2) applying a 60 Hz denoising notch filter to the raw timeseries data before the spectrograms were created since 60 Hz noise is commonly present in EEG data. Each EEG file resulted in 21 channels of data with channel referencing performed as described in the.lbl file associated with each.edf file. An equal number (2,915) of randomly selected 90-s EEG samples with background activity were selected for testing.

#### Experiments to Characterize ESC Classification Performance as a Function of the Amount of Training Data

Multiple training sets were created by selecting ECoG records from 10 to 80 patients in increments of 10 patients. Each training dataset had an equal distribution of patients with few to many ECoG channels to approximate the availability of data in the real-world. Eight of the 113 patients had no ECoG channels with seizure labels and were not included in these experiments. This was done to avoid creating training datasets (especially the ones with data from small number of patients) with highly skewed number of examples of seizure and non-seizure classifications. The remaining 105 patients with ECoG channel labeling were divided into 5 bins based on the number of labeled ECoG channels available for the patient. Each bin contained 21 patients, where bin 1 had patients with the fewest labeled ECoG channels and bin 5 had the patients with the most labeled ECoG channels. Patients for each training set were equally (and randomly) selected from all 5 bins. For example, the training dataset with 10 patients had 2 patients from each of the 5 bins, and the training dataset with 80 patients had 16 patients from each bin. To create incrementally larger training datasets, the same patients from the prior smaller datasets were retained and new patients were added.

The validation dataset for each training dataset was created by randomly sampling from the remaining (non-training) patients in each of the 5 bins equally. Each validation set contained the greater of five patients or 20% of the number of patients in the training dataset. For example, there were 5 patients in the validation datasets for the 10 and 20 patient training datasets, and 6 and 8 patients in the validation datasets for the 30 and 40 patient training datasets, respectively. Table 1B and Supplementary Table 2 shows the number of patients and ECoG channels in each of the 5 folds for each level of patient split.

**TABLE 1B:** Average number of ECoG channels (seizures and non-seizures) in training and validation datasets (and number of ECoG records in the test dataset) used in experiments for characterizing seizure classification accuracy as a function of number of patients’ data used for training.

**Training**			**Validation**			**ECoG record-level testing**		
**# of pts**	**Average # of SZ ECoG channels across 5 folds**	Average # of NSZ ECoG channels across 5 folds*	**# of pts**	**Average # of SZ ECoG channels across 5 folds**	**Average # of NSZ ECoG channels across 5 folds**	**# of pts**	**# of SZ ECoG records in the 5 folds**	**# of NSZ ECoG records in the 5 folds**
10	14,469	23,263	5	5,229	7,717	80	262	333
20	26,064	57,695	5	7,037	15,701	80	262	333
30	39,356	87,258	6	8,441	19,504	80	262	333
40	51,730	113,262	8	10,873	13,212	80	262	333
50	67,034	133,792	10	13,733	25,686	80	262	333
60	80,767	159,479	12	15,352	25,598	80	262	333
70	94,769	181,877	14	16,938	39,042	80	262	333
80	108,277	214,530	16	20,656	37,438	80	262	333

*The averages are computed across the 5 folds for each patient-level split. Numbers of training and validation ECoG channels in each fold for each patient-level split is shown in Supplementary Table 2. ECoG = electrocorticographic; NSZ = non-seizure; pts = patients; SZ = seizure. Shown here is the total number of non-seizure ECoG channels available in the training dataset. Note that the non-seizure ECoG channels were randomly downsampled to match the number of seizure ECoG channels to create balanced 50/50 class splits for training.*

All trained models were tested on the same set of 262 seizure and 333 non-seizure ECoG records from the 80 patients independently labeled by an epileptologist.

### Model Architectures, Training Hyperparameters, and Training Hardware

**FIGURE 5:**
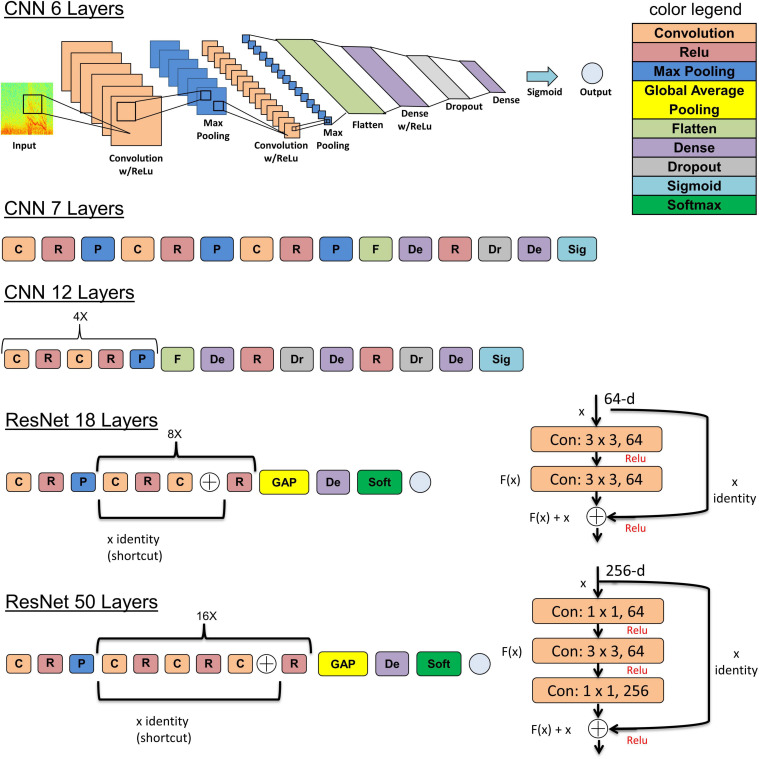
The 5 model architectures used in this study. 6, 7, and 12 layer convolutional neural networks (CNN) models were trained and tested in this study along with 18 and 50 layer ResNet models. Inspiration for the CNN model architectures was derived from a wide range of sources including https://tinyurl.com/y76stqkc, while the ResNet model architectures are available on the Keras webpage: https://keras.io/api/applications/.

The five deep learning models evaluated and are summarized graphically in Figure 5. The original ResNet50 and ResNet18 (available for download from keras.applications) were modified by replacing the final 196 neuron dense layer with a 2 neuron dense layer. This was done to adapt the 18 and 50 layer ResNet models for the 2 class (seizure and non-seizure) classification task described in this paper. Training was performed for a maximum of 70 epochs with a learning rate of 10^–6^ for the 6, 7, and 12 layer CNNs or 10^–7^ for ResNets (18 and 50 layers) with a learning rate decay factor of 0. The choice of learning rate and learning rate decay factor was made after experimenting with a range of values in preliminary experiments. Learning rates higher than those listed above resulted in drastic fluctuations in training and validation performance indicating undesirable divergent behavior in the loss function, and those below the above listed values resulted in very slow, suboptimal training. Initial experiments with fine-tuning the training parameters of only the final few layers of the ResNet models with the initial layers retaining pretrained weights and biases from the imagenet dataset demonstrated substantially worse classification performance on the test dataset, compared to training the parameters in all layers. Hence, the choice was made to fine-tune the weights and biases of all layers. A training, validation and test batch size of 32 was used with all models, and models were trained with the Adam and Nadam optimizers. Training was stopped earlier than 70 epochs, if <0.1% improvement in validation accuracy was observed over 10 consecutive training epochs. The trained model at the epoch number which produced the highest validation accuracy was selected for testing. Keras v2.2.2 with Tensorflow v1.10.1 backend on an on-premise Ubuntu 16.04 machine with two NVIDIA GeForce GTX 1080 Ti GPUs was used for running the experiments described previously in Methods section “Experiments to Test the ECoG-Channel Level and ECoG-Record Level Classification Performance of Trained ESC Models.” Keras v2.3.1 with Tensorflow v2.1.0 on Compute Engine Virtual Machines (machine type: n1-standard-4) with NVIDIA Tesla K80 GPUs on the Google Cloud Platform was used for running the experiments described in 2.6.4. Training was enabled on all layers of all model architectures used in this study.

### Trained Model and Code Availabilty

The trained deep learning models, and ECoG pre-processing code described in this publication may be made available to researchers for academic use. Requests sent to the research-requests@neuropace.com will be reviewed in accordance with NeuroPace data sharing policy and guidelines for requesting support from NeuroPace for research. Key lines of python code for pre-processing ECoGs are made available in the Supplementary Material.

## Results

### Semi-Supervised Labeling of ECoG Records

**FIGURE 6:**
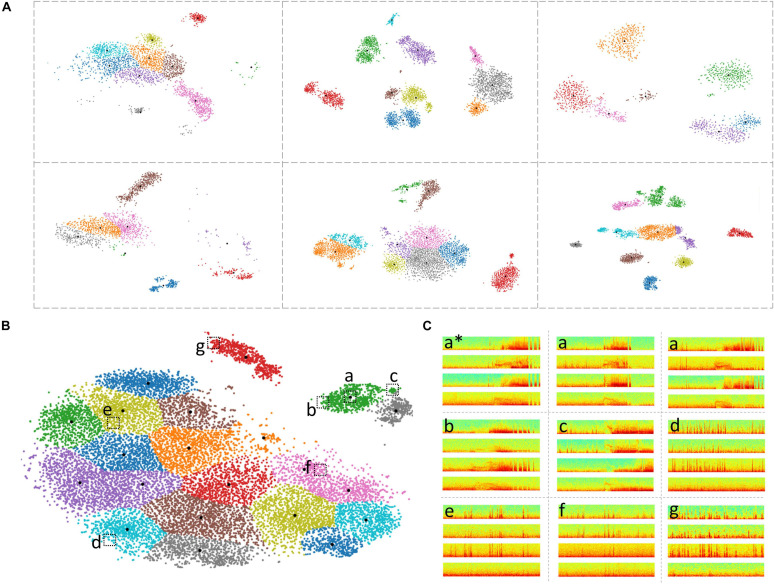
Clustering of ECoG records in patient-specific 2D embedding spaces. **(A)** 2-dimensional embeddings of long episode (LE) electrocorticographic (ECoG) records from six example patients included in this study. Different numbers and patterns of clusters were observed in the patient-specific 2D embedding spaces. **(B)** 2D embeddings of 17,422 LE ECoG records from the patient with the most ECoG records included in this study. Only the green and gray clusters in the top right of the embedding space contained electrographic seizures. **(C)** Example ECoG records from different locations in the 2D embedding space shown in panel **(B)**. ECoG record denoted with ‘a*’ is the centroid ECoG record within the green cluster in panel **(B)**. ECoG records denoted with ‘a’ are two ECoG records that were closest to the centroid ECoG (a*). ECoGs records b and c are two example ECoG records further away from the centroid in the same green cluster. Three example ECoG records (d-f) from other areas of the embedding space contained interictal activity. ECoG record (g) is from a cluster (red cluster in panel **B**) that exclusively contained short (<70 s) interictal ECoG records.

Different patterns, sizes and/or numbers of LE ECoG record clusters were obtained for each patient by the 2D embedding and clustering process (Figure 6A). The median number of clusters obtained with the BGM method was 9 (range: 2-45) with a median of 161 (range: 1-1624) ECoG records in each cluster. While in most cases, sensible cluster identification was obtained with the patient-specific 2D embeddings, in some cases more dispersed LE ECoG record clusters were observed (example orange and purple clusters in Figure 6A bottom-right panel).

Based on *ad hoc* analyses and visual inspection, two primary factors appeared to be responsible for LE ECoG record clustering. One factor was the type of electrographic activity in the ECoG record and the other factor was the ECoG record length. Electrographic activity indicative of seizures tended to produce discrete clusters. Within a patient, multiple seizure clusters were often apparent and distinguishable by seizure morphology and the presence of seizures on different channels. Typically, ECoG records <70 s in length tended to form separate clusters. When electrographic seizures were captured in these short ECoG records, they often clustered separately but in the vicinity of other clusters containing short ECoG records. Since only a minority of LE ECoG records captured with the RNS System were short in length, clusters with short ECoG records were an uncommon occurrence and did not substantially lengthen the manual labeling effort. Similarly, in some patients, ECoG records longer than 90 s (typically 180 s) were captured and in those cases, ECoG records also tended to form separate clusters similar to the short ECoG records. With the clustering tool described above, channel-level manual labeling of 137,985 ECoG records from 113 patients took approximately 320 h. The time required to label all of a patient’s ECoG records depended on the number of records and variability within clusters. The median labeling time was 1.5 h, while for some patients it took as little as <15 min or as long as 3 days. These time estimates include the time spent manually examining all cluster centroid ECoG records in time and spectral domains, assigning each ECoG channel to one of six labels (‘ictal,’ interictal,’ ‘baseline,’ ‘noise,’ ‘low voltage fast only,’ and ‘unsure’) and manually reviewing the pre-assigned (based on the centroid ECoG labels) channel labels for every member of each cluster. As described in the Methods section, if the pre-assigned channel labels did not apply to a given ECoG channel, it was manually corrected after consulting with additional reviewers as needed. The ML-assisted labeling and verification process was faster in patients where most clusters contained similar ECoG records compared to other patients with more ECoG record variability within clusters. In patients with highly similar ECoG records within clusters, usually associated with stereotypical seizure and interictal activity, the entire labeling and verification process took 15 mins or less (for ∼1,200 ECoG records on average). However, it was more common for 10-20% of the ECoG channels within a cluster to require label correction. ML-assisted labeling of 17,422 long episode ECoG records, the most from any patient (Figure 6B) took only 2 h. In this patient, only two out of the 21 clusters contained electrographic seizures. Figure 6C shows nine example ECoG records embedded in the 2D space in panel **(B)**. ECoG records (a) closer to a cluster centroid ECoG (a^∗^) looked more similar to the centroid ECoG compared to ECoG records (b,c) further away from the cluster centroid. This trend was generally seen in all clusters in all patients. Further, ECoG records from other clusters (d-f) looked very different from ECoG records in the green cluster (a-c).

Agreement between an independent expert labeller and author WB on 595 ECoG records from 80 held-out patients (see Methods section “Labeling of ECoG Records”) was at 98.3%. Eight and two ECoG records respectively labeled as seizures and non-seizures by the expert did not agree with the labels provided by author WB.

### ECoG Channel-Level and ECoG Record-Level Classification Performance of Trained ESC Models

#### Model Performance on ECoG Channels From 20% Held-Out Patients

**FIGURE 7:**
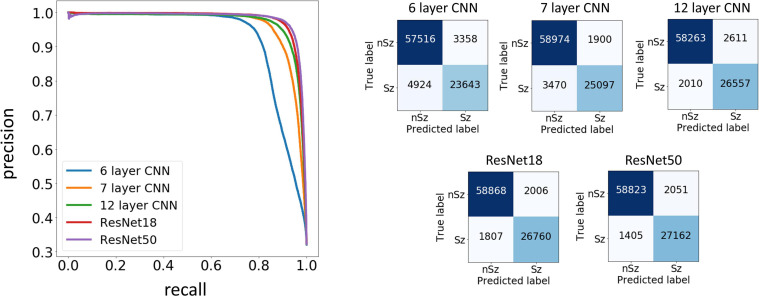
Precision Recall curves and confusion matrices for one example data fold. The trained models assigned a seizure or non-seizure label to individual electrocorticographic (ECoG) channels, along with class prediction probabilities. The precision recall curves **(left)** show the precision or positive predictive value on the *y*-axis, and recall or model sensitivity on the *x*-axis for different thresholds of prediction probabilities, for each of the five model architectures trained in this study. The confusion matrices **(right)** show the number of ECoG channels of each classification (seizure and non-seizure) correctly and incorrectly predicted by the trained models.

Test performances improved with increased depth of models used for training, with the deepest trained model (ResNet50) producing the highest precision and recall values among the five model architectures trained. Figure 7 shows the precision recall curves and confusion matrices for the five CNN models for a randomly chosen data fold. F_1_ scores and test accuracies were generally higher with the Nadam optimizer compared to the Adam optimizer and are shown in Table 2. The corresponding values with the Adam optimizer are shown in Supplementary Table 1. While the ‘sz’ (seizure) class classification accuracy of individual ECoG channels drastically increased with depth of training models, the increase in accuracy in the ‘nsz’ (non-seizure) class was moderate (Figure 7B and Table 2). Increases in overall accuracies of 3.8% and 3.26% were observed when the depth of the training model was increased from 6 to 7 layers, and 7 to 12 layers respectively. However, accuracy increases of only 0.51% and 0.28% were observed when model depth was further increased from 12 layers to 18 layers, and 18 layers to 50 layers respectively. Similar trends were observed with F_1_ scores, indicating that the point of diminishing returns relative to model depth is around 12 layers. Among the five different model architectures studied, the ResNet50 model had the highest F_1_ score (94.26%) and class-balanced accuracy (95.72%), with 97.17% non-seizure class accuracy and 94.26% seizure class accuracy. In addition to producing higher test performances, deeper (≥12 layers) models also resulted in less fold to fold variation in F_1_ scores and accuracies compared to the shallower models (2.9%, 1.5%, 0.6%, 0.4% and 0.4% standard deviation in overall accuracy values for the 6 layer, 7 layer, 12 layer CNN, ResNet18 and ResNet50 respectively), indicating that the shallower models not only underfit the training data, but were also less robust compared to the deeper models.

**TABLE 2:** Test performance on held-out ECoG channels and expert labeled ECoG records.

**Model**	**Fold**	**Performance on ECoG channels from 20% held-out patients**				**Performance on ECoG records from 80 expert labeled held-out patients**			
		**Test accuracy %**			**F_1_ score**	**Test accuracy %**			**F_1_ score**
		**Overall**	**NSZ Class**	**SZ Class**		**Overall**	**NSZ Class**	**SZ Class**	
**6 layer** LR: 10^–6^ Opt: Nadam	1	90.16	93.59	86.74	89.37	86.32	92.49	80.15	84.51
	2	88.62	94.48	82.76	85.10	84.21	93.99	74.43	81.76
	3	83.03	93.91	72.14	77.72	81.00	89.49	72.52	78.03
	4	89.79	92.62	86.97	84.08	83.68	97.90	69.47	80.71
	5	87.80	89.93	85.66	84.15	89.85	94.59	85.11	88.67
	**Avg**	**87.88**	**92.90**	**82.86**	**84.08**	**85.01**	**93.69**	**76.34**	**82.73**
**7 layer** LR: 10^–6^ Opt: Nadam	1	91.84	95.20	88.48	91.22	90.35	95.20	85.50	89.24
	2	92.37	96.88	87.85	90.34	90.60	87.69	93.51	89.42
	3	89.65	97.55	81.75	87.34	90.15	95.20	85.11	89.02
	4	93.61	95.24	91.98	89.83	91.97	97.30	86.64	91.16
	5	90.89	93.21	88.58	88.28	92.64	95.20	90.08	91.83
	**Avg**	**91.67**	**95.62**	**87.73**	**89.40**	**91.14**	**94.11**	**88.17**	**90.13**
**12 Layer** LR: 10^–6^ Opt: Nadam	1	95.15	96.51	93.79	94.84	95.68	96.70	94.66	95.20
	2	94.34	95.71	92.96	92.00	93.48	94.59	92.37	92.72
	3	94.28	97.12	91.46	92.44	94.19	92.19	96.18	93.33
	4	95.62	97.29	93.95	93.37	95.13	93.69	96.56	94.40
	5	95.24	98.27	92.20	94.43	95.19	96.10	94.27	94.64
	**Avg**	**94.93**	**96.98**	**92.87**	**93.41**	**94.73**	**94.65**	**94.81**	**94.06**
**RN18** LR: 10^–7^ Opt: Nadam	1	96.05	97.03	95.07	95.79	93.82	90.69	96.95	92.87
	2	95.19	96.71	93.68	93.35	91.88	85.29	98.47	90.69
	3	95.13	97.98	92.27	93.79	93.97	90.99	96.95	93.04
	4	95.07	95.67	94.47	91.66	94.99	91.89	98.09	94.14
	5	95.76	97.22	94.30	94.65	95.52	92.19	98.85	94.70
	**Avg**	**95.44**	**96.92**	**93.96**	**93.85**	**94.04**	**90.21**	**97.86**	**93.09**
**RN50** LR: 10^–7^ Opt: Nadam	1	96.07	97.09	95.04	95.81	94.57	92.19	96.95	93.73
	2	95.86	96.63	95.08	94.02	91.91	90.69	93.13	90.88
	3	95.14	98.04	92.24	93.84	92.89	91.89	93.89	91.96
	4	95.57	96.32	94.82	92.63	95.00	95.50	94.66	94.48
	5	95.94	97.74	94.14	95.01	93.26	89.19	97.33	92.22
	**Avg**	**95.72**	**97.17**	**94.26**	**94.26**	**93.54**	**91.89**	**95.19**	**92.65**

*ECoG = electrocorticographic; NSZ = non-seizure; SZ = seizure. Bold values are average of 5 folds.*

ECoG length affected classification accuracy when comparing short (<80 s), regular (90 s) and long (>100 s) records. With the ResNet50 model, the average classification accuracies on short (92.7%) and long (94.1%) ECoG channels were significantly lower (*p* < 0.05, Wilcoxon rank sum test) compared to classification accuracy on 90-s (96.4%) ECoG channels. Similar trends in classification performance with short, long and regular-length ECoG channels were observed with all model architectures trained. The average classification performance of the ResNet50 models on short ECoG channels was slighty better when preprocessing was performed by duplicating and concatenating the starting portion of the ECoG (92.7%), when compared to zero-padding (91.8%), although this difference was not statistically significant. The average classification performance was slightly better (statistically non-significant) when the stimulation artifact rejection step was applied (95.7%), compared to when it was not applied (95.5%). Additionally, the classification accuracy was significantly better (*p* < 0.05, Wilcoxon rank sum test) when model training was done with the spectrogram images saved in the ‘jet’ colormap (95.7%), compared to spectrogram images saved in the ‘grayscale’ colormap (94.9%).

#### Model Performance on ECoG Records From 80 Expert-Labeled Held-Out Patients

As described in the Methods section, an ECoG record was classified as a seizure if any of the 4 channels were classified as a seizure by the trained ESC models. A non-seizure label was assigned only if all 4 channels were classified as non-seizures. A random binary classifier (with 50% chance of classifying an ECoG channel as a seizure) would classify a 4-channel ECoG record as seizure 93.75% of the time, and it would classify a 4-channel ECoG record as non-seizure only 6.25% of the time. Hence, a random classifier would produce extremely skewed class-specific accuracies guessing seizure ECoG records correctly most of the time, while having a very poor non-seizure class accuracy. In comparison, all trained models produced more or less balanced seizure and non-seizure class accuracies (as shown in Table 2B), demonstrating successful training, with the test performance generally improving with depth. A significant (*p* < 0.01, Wilcoxon rank sum test) increase of 9.72% in overall accuracy and an 11.32% increase in F_1_ score was observed between the 6 layer CNN and the 12 layer CNN models, and a non-significant difference in test performance (< 1.41%, p > 0.05, Wilcoxon rank sum test) was observed between the 12 layer CNN and ResNet50-based model. Similar to observations with ECoG channel level test accuracies, substantial improvements in seizure class accuracies were observed with increased CNN model depths, while the non-seizure class accuracies remained relatively constant.

Training and validation curves for each type of model are shown in Supplementary Figure 1. Training and validation accuracies with the deeper ResNet50 and ResNet18 models increased rapidly over the first few epochs with the condition for early stopping (< 0.1% improvement in validation accuracy observed over 10 consecutive training epochs) applying substantially earlier than with shallower models.

#### Error Analysis

**FIGURE 8:**
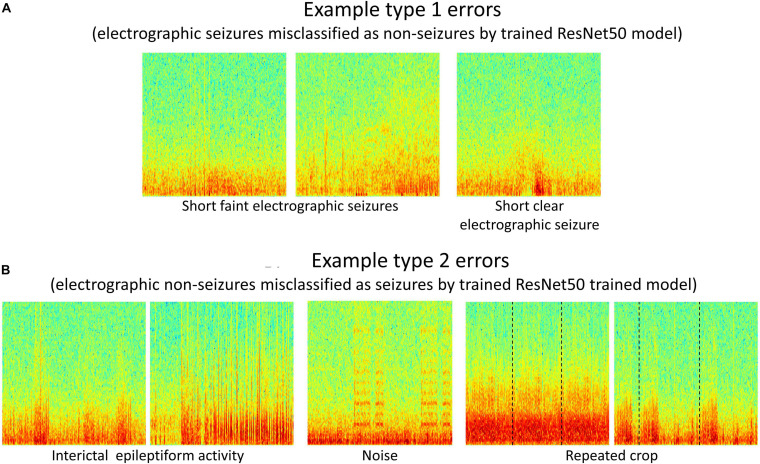
**(A,B)** Example type 1 and type 2 errors by a trained ResNet50 model. Type 1 errors typically included short faint electrographic seizures, while type 2 errors typically included long trains of high amplitude interictal events, noise and repeated crops created during pre-processing electrocorticographic (ECoG) channels in cases where length was shorter than 80 s.

Type 1 (False negative) and Type 2 (False positive) error rates for the representative data fold shown in Figure 7 are 17% and 6% for the 6 layer CNN model, 12% and 6% for the 7 layer CNN model, 7% and 4% for the 12 layer CNN model, 6% and 5% for ResNet18 model, and 6% and 3% for the ResNet50 model respectively. In all data folds, the deepest ResNet50 model produced the least percentage of type 1 and type 2 errors. Examination of errors showed that between 10-15% of the misclassified ECoG channels could be attributed to labeler error and were not model performance errors. The remaining errors were due to model performance. A few example ResNet50 classification errors are shown in Figure 8. Seizure spectrograms with only faint frequency bands, lower amplitude changes, or short durations (around 10 s) were sometimes misclassified (Figure 8A). Also, non-seizure spectrograms were sometimes misclassified when they contained cropped interictal ECoG channels, resulting in short bursts of high frequency or amplitude activity (typically found in electrographic seizures) being repeated for longer than 10 s (Figure 8B). Finally, if ECoGs longer than 100 s contained multiple long episode triggers, the last one would be used for creating the spectrogram image. This infrequently resulted in the main portion of the seizure being cropped out of the resulting image, resulting in the ECoG channel getting erroneously labeled as non-seizure.

#### Saliency Maps

**FIGURE 9:**
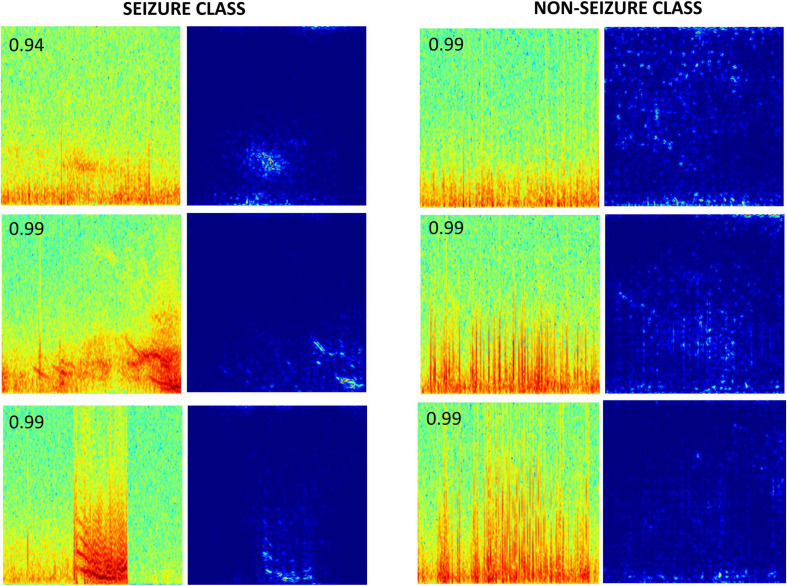
Saliency maps of a few correctly classified seizure and non-seizure class ECoG channels by the ResNet50 ESC model. The number on the top left corner in each spectrogram image is the prediction probability of the model for the respective class.

Gradient-based saliency maps associated with the final fully connected layer of the trained ResNet50 model are shown in Figure 9. It appears that in seizure class, horizontal and diagonal spectral power bands within the seizure activity are generally highlighted, confirming that the neural network’s seizure classifications are indeed based on seizure activity, and is not a consequence of irrelevant feature learning. In comparison, non-seizure class predictions had scattered background activation patterns often associated with interictal spiking activity.

#### Model Generalizability to EEG Datasets Not Captured With the RNS System

On the TUH EEG Seizure Corpus, the ResNet50 ESC models had an average overall classification accuracy of 70.1% with an average F_1_ score of 69.5%. The average seizure class classification accuracy was 68.2%, while the non-seizure class classification accuracy was 72%. In other words, 926 out of 2,915 seizure spectrograms were missclassified as non-seizures, while 1,989 were correctly classified. Similarly, 817 out of 2,915 non-seizure spectrograms were missclassified as seizures, while 2,089 were correctly classified.

Error analysis revealed that the most common explanation for the seizure and non-seizure misclassification in the TUH EEG dataset was the presence of various types of noise in the raw EEG data, with the classification performance generally degrading with increased levels of noise in the EEG spectrograms. A few examples of correctly and incorrectly classified EEG spectrograms from the TUH EEG Seizure Corpus are shown in Supplementary Figure 2.

### ESC Classification Performance as a Function of Amount of Training Data

**FIGURE 10:**
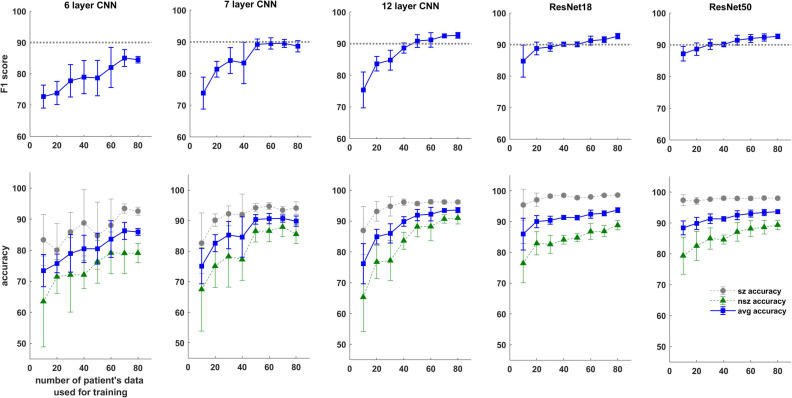
F_1_ score and test accuracy of CNN and ResNet models trained with data from increasing numbers of patients. Convolutional neural networks (CNN) and ResNet models were tested on expert labeled electrocorticographic (ECoG) records from 80 patients. A dotted line is drawn at F_1_ score value of 90% for reference.

Trends in F_1_ scores and class-balanced accuracies vs amount of training data are shown in Figure 10 and Table 3. Models were tested on expert labeled ECoG records from 80 patients. The results show that training data from a minimum of 30 patients is required to achieve > 90% generalizability in new patients. With the 6 layer CNN model, none of the 8 patient splits achieved > 90% accuracy or F_1_ score. Training data from 50 patients were required to achieve F_1_ scores and accuracies of over 90% with the 12 layer CNN models. On the other hand, the deeper ResNet18 and ResNet50 models performed better, requiring training data from fewer patients i.e., 40 and 30 patients respectively, to achieve ECoG record-level classification accuracies of >90% in new patients. Deeper training models and larger training datasets produced lower fold to fold variation in performance metrics (see standard deviation values in Table 3 and Supplementary Table 3), compared to shallow models and smaller training datasets.

**TABLE 3:** F_1_scores (mean ± standard deviation) as a function of number of patient’s data used for training.

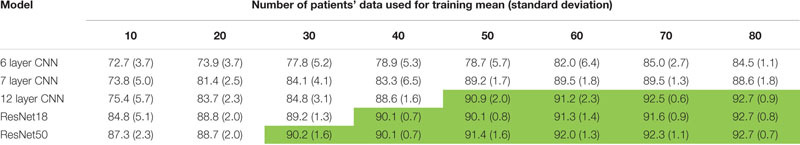

*CNN = convolutional neural networks. Cells with F_1_ scores ≥ 90% are filled with green color.*

## Discussion and Conclusion

The work in this paper is novel and significant for several reasons. First, it describes a semi-supervised technique for labeling large ECoG datasets. This can be an important step for building supervised machine learning models on large datasets, which is becoming increasingly common in the heathcare domain. Second, it shows that despite the heterogeneous nature of electrographic seizures, robust electrographic seizure detection models can be built that classify ambulatory ECoG channels in new patients with over 95% classification accuracy. Third, it validates the method of converting time-series ECoG data to spectrogram images for the purpose of CNN-based ECoG classification models. Finally, it shows that with good CNN architecture selection, data from as few as 10 patients can produce cross-patient electrographic seizure classification accuracies of 88% (F_1_ score 87%), while a minimum of 30 patient’s labeled ECoG records may be required for achieving a classification accuracy of over 90%.

The prohibitive task of labeling a large ECoG dataset was made manageable with the aid of an ECoG record clustering tool. This tool enabled the manual labeling of ∼138,000 ECoG records in 320 h. Using only 2 labels instead of 6 (as was the case in this study) would have resulted in even faster labeling. It should be emphasized that the unsupervised ECoG record custering step was followed by a manual label validation step in which pre-assigned labels given to every ECoG record were manually verified, and corrections were made as necessary. However, without the use of such an ECoG clustering tool for pre-labeling ECoG records, a conservative estimate for labeling ∼138,000 4-channel ECoG records is around 1533 h (at ∼10 s on average for labeling each ECoG channel), not accounting for delays caused by labeler-fatigue. Thus, use of the ECoG record clustering tool to speed-up labeling of a large ECoG dataset was key to the success of this project.

A goal of this study was to manually label the large NeuroPace ECoG dataset for the purpose of building an electrographic seizure classifier. A simple, ‘out-of-the-box’ technique that produced reasonably good within-patient ECoG clustering was desired, and hence the pre-trained GoogLeNet Inception-V3 model which can be applied to 3-color channel image data was chosen as feature extractor, after experimenting with a few different pre-trained CNN models. Dimensionality reduction was then performed with PCA and t-SNE. Even though t-SNE is a technique not known for preserving intercluster distances, in our case, where ECoG labeling was done on a per-cluster basis, preserving intercluster distances was not a priority and did not negatively impact the manual labeling process. In applications where preserving inter-cluster distances is of importance, other more recently developed techniques such as UMAP could be used instead (McInnes et al., 2018). In future studies, models specifically trained on ECoG records, such as autoencoders trained on unlabeled ECoG records (Tsinalis et al., 2016), or CNN models trained on auxiliary tasks such ECoG classification (i.e., similar to the ESC models trained in this study) may be used as feature extractors to improve ECoG record clustering performance. Alternatively, deep ranking models trained using the triplet loss function with the goal of learning optimal embedding functions, may be used (Wang et al., 2014). Nevertheless, as shown in this present study, the use of pre-trained CNN models for feature extraction followed by dimensionally reduction proved to be adequately useful, and as far as we know this is the first study to demonstrate the usefulness of transferring pre-trained CNN weights to spectrogram images of brain recordings for clustering brain data. Such a transfer learning technique is likely to translate effectively to other types of physiological data (Salem et al., 2018).

Long episode ECoG records make up over 50% of all ECoG records captured by the NeuroPace RNS System and contain a mix of baseline, interictal and ictal activity. Out of the 414,933 manually labeled channels from 137,985 long episode ECoG records, 140,183 were given the seizure label, and 274,750 were given one of five non-seizure labels. Since a sufficient number of ECoG channels with the non-seizure label was available from only labeling the long episode ECoG records, in the interest of minimizing manual labeling time, it was deemed unnecessary to label other types of ECoG records captured by the RNS System (such as scheduled records and records triggered by a patient applied magnet) for the purpose of training ESC models. The other types of ECoG records contain a much lower proportion of seizures and many more non-seizure records. Since balanced numbers of seizure and non-seizure ECoGs were used for training, it is unlikely that including these ECoGs in the training sets would have improved performance.

Five types of model architectures were trained and tested. Three of them involved convolutional layers, max pooling, and dense layers, connected linearly, and two of them involved residual connections, as shown in Figure 5. Increasing the depth of CNNs from 6 to 7 layers, and from 6 to 12 layers resulted in improvements in ECoG channel-level accuracies of 3.8% and 7.1%, respectively, with an accompanying decrease in fold-to-fold performance variability. Since issues such as vanishing and exploding gradients can manifest from pushing a neural network too deep, the ResNet architectures, which have residual connections to mitigate the above mentioned problems, were used when expanding beyond 12 layers. Modest improvements in accuracy were observed between the 12 layer CNN and the 18 layer ResNet-based (0.51% improvement), and between the 12 layer CNN and 50 layer ResNet-based models (0.79% improvement) suggesting that the point of diminishing returns with respect to model depth/complexity may be around 12 layers. When selecting a model for incorporation of an ESC in embedded systems, the 12 layer CNN may be a better choice compared to the ResNet-based models because of its substantially smaller computational demands while having only slightly worse classification performance. It should be noted, however, that since the models described here were optimized for classifying 90-s ECoG records, pipeline modifications and model revisions may be necessary to identify seizures in a continuous data stream. Additionally, the trained models may need to be converted to compressed, lighter formats (the Tensorflow Lite format, for example) for real-time model inference in embedded systems with limited compute capability.

The classification performance of the ESC trained models were only slightly worse on short (ResNet50 model accuracy: 92.7%, 12 layer CNN model accuracy: 91.1%) and long (94.1%, 93.3%) ECoG channels compared to the classification performance on 90-s ECoG channels (96.4%, 95.4%). This small discrepancy in performance was expected given that ∼67% of the data used in this study were the regular-length (i.e., 90-s) ECoG channels. Even though the trained ESC models performed best on regular-length EcoG channels, the classification performances on short and long EcoG channels were still very impressive making the trained models applicable to EcoG channels of all durations captured with the RNS System (maximum EcoG storage length is 240 s, minimum is 30 s).

A few different hyperparameters such as the initial learning rate, the learning rate decay factor, the optimizer type were tuned empirically. Preliminary tests showed that initial learning rates of 10^–6^ for the shallower (6,7, and 12 layer) CNNs, and 10^–7^ for the deeper ResNet-based architectures, with a learning rate decay of 0, produced the best training and validation accuracies among the parameter values tested, so these values were used for all experiments reported in this paper. The choice of weight initialization method (Xavier vs ImageNet weights) did not make a difference on the model’s final training performance, so ImageNet weight initialization was used where available (i.e., for the ResNet models), and Xavier initialization was used in other cases. Additionally, the choice of gradient descent optimization algorithm, i.e., Adam vs Nadam, did not make a substantial difference to the models’ performance, although the Nadam (which is Adam with Nesterov Momentum) optimizer produced slightly better results. Overall, model training and testing performance seemed to be robust to small variations in hyperparameter selections.

Gradient-based saliency maps were examined to confirm that the features learned by the trained ESC models are relevant to the classification task. Saliency maps associated with seizure classifications, generally had pixels associated with horizontal and diagonal seizure power bands highlighted, while saliency maps associated with non-seizure classifications had pixels associated with any interictal spiking activity occasionally highlighted. Finally, tests were run to confirm that the choice of training platform (virtual machines on the Google Cloud Platform vs on-premise machines) did not affect model training and testing performances.

Although model architectures and hyperparameters such as the learning rate, optimizers and spectrogram colormaps (which are often considered to be among the most important hyperparameters) were experimented with, the hyperparameter search space is enormous. In future experiments, neural architecture search methods such as AutoML on Google Cloud Platform will be used to further improve classification accuracies. Additionally, data from only 113 patients were used for training, validation and testing in this study. Manually labeling data from all 256 RNS study patients and using data from additional patients for training may lead to further improvements in model performance.

The trained ESC models performed substantially better than chance (70.1%; chance level is 50%) in classifying spectrogram images of EEG data in the TUH EEG Seizure Corpus (an EEG dataset not captured with the RNS System). Error analysis revealed that the most common explanation for misclassification was various types of noise present in the raw EEG data, with the classification performance generally being much better on EEG datasets with little/no noise, compared to nosier datasets. This was expected given that the RNS System ECoG data used to train the CNN models were practically noise-free. The only type of denoising applied by us to the TUH EEG data was a 60 Hz notch filter. The addition of other denoising steps, while outside the scope of this paper, could potentially lead to substantially better classification outcomes. The fact that the ESC models trained on ECoG data captured with the RNS System could classify seizures in EEG datasets captured with markedly different lead types, electrode configurations and recording electronics demonstrates the transferability of preprocessing methods and trained models across different types of time-series brain recording datasets. Fine-tuning weights and biases in final layers of neural networks trained on large ECoG/EEG datasets (such as the RNS System data used in this study) with smaller labeled time-series brain datasets from a different source could be a potential strategy for applying deep learning classification/regression models to datasets with limited training data. Such a technique is frequently used for solving computer vision problems (Shin et al., 2016).

As expected, the performance of trained models continued to improve with the amount of training data used. In all patient-level splits the deepest model (ResNet50 model) produced the best results while shallowest model (CNN 6 layer) produced the worst. Class-balanced accuracies were >90% when the ResNet50 model was trained with labeled ECoG records from 30 patients. Surprisingly, training ResNet50-based models with labeled spectrogram images from just 10 patients produced a mean ECoG record classification accuracy of ∼88% in new patients suggesting that the deeper ResNet50 models can effectively learn electrographic seizure signatures even with limited training examples, and can successfully generalize the learnings to ECoG records from new patients. The shallow 6 layer CNN, on the other hand, only produced a classification accuracy of 73% with 10 patient’s training data. These findings suggest that for EEG/ECoG classification tasks, it may be beneficial to train deep CNN architectures (such as the ResNet50 architecture) even in cases where data from only a limited number of subjects are available, and that training experiments should not be limited to shallow CNN models as has mostly been the case in the past (Roy et al., 2019).

Future experiments are needed to study the relationship between the distinct number of electrographic seizure patterns used for training and cross-patient classification accuracy. In our experiments to characterize model performance as a function of number of patient’s data used for training, a substantial number (∼14,500 per class or ∼29,000 in total) of seizure and non-seizure spectrogram images were available from just 10 patients. Since some patients have multiple seizure foci and seizure waveforms can change over time, each patient likely contributed a few different seizure patterns for training. This is because seizures may originate in different brain areas with different seizure waveforms, and because ECoG records used in this study cover an average of 7.5 years per patient. This variety could explain why models built with just 10 patients’ data generalized to new patients with > 88% accuracy. Repeat analyses with data captured from small numbers of patients over shorter periods will provide additional insight into the minimum number of seizure examples required for good performance.

About 34% of labeled ECoG channels used in this paper belonged to the seizure class, while the remaining 66% of ECoG channels belonged to the non-seizure class. Class balancing of seizure and non-seizure class ECoG channels was performed only in the training datasets and not in the test datasets. This was done to compute the test performance on realistic distributions of ECoG records captured with the RNS System. Hence, the precision (positive predictive value), recall (sensitivity) and F_1_ scores (harmonic mean of precision and recall) reported in this paper were computed on imbalanced datasets with about twice the number of non-seizure examples as seizure examples (see Table 1). However, these metrics do not reflect model performance on continuous ECoG records. The RNS System captures data intermittently with data capture biased toward abnormal ECoG activity. In this paper, a training approach that produced high cross-patient test classification performance on such intermittently captured ECoG records was chosen. Although training was not performed with the goal of applying the models to continuous ECoG records, extrapolating the results (97.17% non-seizure accuracy and 94.26% seizure accuracy on 90 s ECoG records; see Table 2) to continuous ECoG records would result in a false positive rate of ∼1.1/h per ECoG channel (with the sensitivity still being high at 94.26%). This is assuming that the continuous ECoG records are cut into 90 s non-overlapping segments and fed into the model for inference. If a patient only has a few seizures per month, this could produce a large number of false positives relative to true positives. Nevertheless, such a generic cross-patient model could still be used in an offline setting to filter out ∼97% of data and present the remaining 3% for further human review. A better strategy, however, might be to alter the training approach to optimize model classification performance on continuous ECoG records. For example, data augmentation techniques could be used to mimic continuous data capture by enriching scheduled ECoGs in a training set of all ECoG record types, and custom loss functions with additional penalties could be used for minority class misclassifications. Such experiments are outside the scope of this paper but certainty warrant further studies.

Error analysis revealed some shortcomings in model performance. In general, seizure ECoG channels with very subtle seizure signals in spectrogram images were sometimes misclassified as non-seizures. Conversely, non-seizure spectrogram images with noise artifact or long trains of high amplitude interictal activity were occasionally misclassified as seizures. Several approaches may be taken to further improve the classification performance of the electrographic seizure detection models. Training ECoG channel classification models with greater spatial context such as including spectrogram images of ECoG activity from adjacent ECoG channels could lead to higher classification accuracy for the primary channel. This would mimic the behavior of a human labeler evaluating faint seizure activity by using activity on adjacent ECoG channels. Additionally, separate CNN or ResNet models could be trained on shorter and longer ECoGs to improve the classification performance of ECoG channels that are outside the typical length range of 90 s. Since relatively few ECoG channels fall outside the typical length range, one way to achieve this could be to fine-tune the final layer(s) parameters of a base CNN or ResNet model trained with 90-s ECoGs for the shorter and longer ECoGs respectively. Alternatively, recurrent neural networks architectures (RNN) or combined CNN and RNN architectures which do not require the input data to be of fixed dimensions may be trained.

In summary, this study demonstrates that converting time-series ECoG records into spectrogram images and using them as input to CNN models can be used to effectively train robust cross-patient seizure classification models. Healthcare tools built using these models may facilitate the physician’s review of EEG data for epilepsy patients, and have the potential to improve clinical outcomes due to improved diagnostic assessments. Additionally, by characterizing the performance of various CNN models as a function of amount of training data, this research provides ECoG/EEG data collection guidance for researchers interested in solving similar ECoG classification problems.

## Data Availability Statement

The data analyzed in this study is subject to the following licenses/restrictions: The trained deep learning models, and ECoG pre-processing code described in this publication may be made available to researchers for academic use. Requests sent to the research-requests@neuropace.com will be reviewed in accordance with NeuroPace data sharing policy and guidelines for requesting support from NeuroPace for research. Key lines of python code for pre-processing ECoGs are made available in the Supplementary Material.

## Ethics Statement

All study protocols were approved by the FDA and the institutional review boards of the participating investigation sites. All participants gave written informed consent. The RNS System Feasibility, Pivotal, and LTT studies are registered on clinicaltrials.gov (NCT00079781, NCT00264810, and NCT00572195). The patients/participants provided their written informed consent to participate in this study.

## Author Contributions

SA designed the study and performed the ECoG clustering experiments, ESC model training and testing experiments, and primarily wrote the manuscript. WB manually labeled ECoG records from 113 patients, performed ESC model training and testing experiments, and error analyses. TT and MM were involved in study and manuscript development. All authors contributed to the article and approved the submitted version.

## Conflict of Interest

WB, TT, and SA have equity ownership/stock options with NeuroPace and was current employee of NeuroPace. MM has equity ownership/stock options with NeuroPace and is currently employed as Chief Medical Officer of NeuroPace.

## Footnotes

1 https://www.mturk.com/

2 https://www.figure-eight.com/

3 https://www.samasource.com/

4 https://www.isip.piconepress.com/projects/tuh_eeg/

## References

[B1] Acharya U. R. Oh S. L. Hagiwara Y. Tan J. H. Adeli H. (2018). Deep convolutional neural network for the automated detection and diagnosis of seizure using EEG signals. *Comput. Biol. Med.* 100 270–278. 10.1016/j.compbiomed.2017.09.017 28974302

[B2] Ansari A. H. Cherian P. J. Caicedo A. Naulaers G. De Vos M. Van Huffel S. (2019). Neonatal seizure detection using deep convolutional neural networks. *Int. J. Neural Syst.* 29:1850011. 10.1142/S0129065718500119 29747532

[B3] Arora A. Lin J. J. Gasperian A. Maldjian J. Stein J. Kahana M. (2018). Comparison of logistic regression, support vector machines, and deep learning classifiers for predicting memory encoding success using human intracranial EEG recordings. *J. Neural Eng.* 15:066028. 10.1088/1741-2552/aae131 30211695 PMC9020643

[B4] Baumgartner C. Koren J. P. (2018). Seizure detection using scalp-EEG. *Epilepsia* 59 14–22. 10.1111/epi.14052 29873826

[B5] Bazil C. W. Pitkänen A. Loeb J. A. Dudek F. E. Bertram E. H. III Cole A. J. (2004). Sleep, sleep apnea, and epilepsy. *Curr. Treat. Options Neurol.* 6 339–345. 10.1007/s11940-004-0033-4 15157411

[B6] Bergey G. K. Morrell M. J. Mizrahi E. M. Goldman A. King-Stephens D. Nair D. (2015). Long-term treatment with responsive brain stimulation in adults with refractory partial seizures. *Neurology* 84 810–817. 10.1212/WNL.0000000000001280 25616485 PMC4339127

[B7] Bruno E. Simblett S. Lang A. Biondi A. Odoi C. Schulze-Bonhage A. (2018). Wearable technology in epilepsy: the views of patients, caregivers, and healthcare professionals. *Epilepsy Behav.* 85 141–149. 10.1016/j.yebeh.2018.05.044 29940377

[B8] Desai S. A. Tcheng T. Morrell M. (2019a). “Transfer-learning for differentiating epileptic patients who respond to treatment based on chronic ambulatory ECoG data,” in *Proceedings of the 2019 9th International IEEE/EMBS Conference on Neural Engineering (NER)*, (New York, NY: IEEE). 10.1109/NER.2019.8717007

[B9] Desai S. A. Tcheng T. K. Morrell M. J. (2019b). Quantitative electrocorticographic biomarkers of clinical outcomes in mesial temporal lobe epileptic patients treated with the RNS§system. *Clin. Neurophysiol.* 130 1364–1374. 10.1016/j.clinph.2019.05.017 31212202

[B10] Engel J. (2011). Biomarkers in epilepsy: introduction. *Biomark. Med.* 5 537–544. 10.2217/bmm.11.62 22003902

[B11] Engel J. Jr. Pitkänen A. Loeb J. A. Dudek F. E. Bertram E. H. III Cole A. J. (2013). Epilepsy biomarkers. *Epilepsia* 54 61–69. 10.1111/epi.12299 23909854 PMC4131763

[B12] Haas S. M. Frei M. G. Osorio I. (2007). Strategies for adapting automated seizure detection algorithms. *Med. Eng. Phys.* 29 895–909. 10.1016/j.medengphy.2006.10.003 17097325 PMC2339717

[B13] Halford J. J. Pressly W. B. Benbadis S. R. Tatum W. O. IV Turner R. P. Arain A. (2011). Web-based collection of expert opinion on routine scalp EEG: software development and interrater reliability. *J. Clin. Neurophysiol.* 28 178–184. 10.1097/WNP.0b013e31821215e3 21399515

[B14] Halford J. Shiau D. Desrochers J. A. Kolls B. J. Dean B. C. Waters C. G. (2015). Inter-rater agreement on identification of electrographic seizures and periodic discharges in ICU EEG recordings. *Clin. Neurophysiol.* 126 1661–1669. 10.1016/j.clinph.2014.11.008 25481336 PMC4439396

[B15] He K. Zhang X. Ren S. Sun J. (2016). “Deep residual learning for image recognition,” in *Proceedings of the IEEE Conference on Computer Vision and Pattern Recognition*, (Las Vegas, NV). 10.1109/CVPR.2016.90

[B16] Hoppe C. Poepel A. Elger C. E. (2007). Epilepsy: accuracy of patient seizure counts. *Arch. Neurol.* 64 1595–1599. 10.1001/archneur.64.11.1595 17998441

[B17] Kerling F. Mueller S. Pauli E. Stefan H. (2006). When do patients forget their seizures? An electroclinical study. *Epilepsy Behav.* 9 281–285. 10.1016/j.yebeh.2006.05.010 16824803

[B18] Kuanar S. Athitsos V. Pradhan N. Mishra A. Rao K. R. (2018). “Cognitive analysis of working memory load from EEG, by a deep recurrent neural network,” in *Proceedings of the 2018 IEEE International Conference on Acoustics, Speech and Signal Processing (ICASSP)*, (New York, NY: IEEE). 10.1109/ICASSP.2018.8462243

[B19] LeCun Y. Bengio Y. Hinton G. (2015). Deep learning. *Nature* 521 436–444. 10.1038/nature14539 26017442

[B20] Macosko E. Z. Basu A. Satija R. Nemesh J. Shekhar K. Goldman M. (2015). Highly parallel genome-wide expression profiling of individual cells using nanoliter droplets. *Cell* 161 1202–1214. 10.1016/j.cell.2015.05.002 26000488 PMC4481139

[B21] Manford M. Fish D. Shorvon S. (1996). An analysis of clinical seizure patterns and their localizing value in frontal and temporal lobe epilepsies. *Brain* 119 17–40. 10.1093/brain/119.1.17 8624679

[B22] McInnes L. Healy J. Melville J. (2018). Umap: uniform manifold approximation and projection for dimension reduction. *arXiv [Preprint].* 10.21105/joss.00861

[B23] Morrell M. J. (2011). Responsive cortical stimulation for the treatment of medically intractable partial epilepsy. *Neurology* 77 1295–1304. 10.1212/WNL.0b013e3182302056 21917777

[B24] O’Shea A. Lightbody G. Boylan G. Temko A. (2018). “Investigating the impact of CNN depth on neonatal seizure detection performance,” in *Proceedings of the 2018 40th Annual International Conference of the IEEE Engineering in Medicine and Biology Society (EMBC)*, (New York, NY: IEEE). 10.1109/EMBC.2018.8513617 30441669

[B25] Regalia G. Onorati F. Lai M. Caborni C. Picard R. W. (2019). Multimodal wrist-worn devices for seizure detection and advancing research: Focus on the Empatica wristbands. *Epilepsy Res.* 153 79–83. 10.1016/j.eplepsyres.2019.02.007 30846346

[B26] Roy Y. Banville H. Albuquerque I. Gramfort A. Falk T. H. Faubert J. (2019). Deep learning-based electroencephalography analysis: a systematic review. *J. Neural Eng.* 16: 051001. 10.1088/1741-2552/ab260c 31151119

[B27] Russakovsky O. Deng J. Su H. Krause J. Satheesh S. Ma S. (2015). Imagenet large scale visual recognition challenge. *Int. J. Comput. Vis.* 115 211–252. 10.1007/s11263-015-0816-y

[B28] Ryapolova-Webb E. Afshar P. Stanslaski S. Denison T. de Hemptinne C. Bankiewicz K. (2014). Chronic cortical and electromyographic recordings from a fully implantable device: preclinical experience in a nonhuman primate. *J. Neural Eng.* 11:016009. 10.1088/1741-2560/11/1/016009 24445430

[B29] Salem M. Taheri S. Yuan J. S. (2018). “ECG arrhythmia classification using transfer learning from 2-dimensional deep CNN features,” in *Proceedings of the 2018 IEEE Biomedical Circuits and Systems Conference (BioCAS)*, (New York, NY: IEEE). 10.1109/BIOCAS.2018.8584808

[B30] Shah V. von Weltin E. Lopez S. McHugh J. R. Veloso L. Golmohammadi M. (2018). The temple university hospital seizure detection corpus. *Front. Neuroinform.* 12:83. 10.3389/fninf.2018.00083 30487743 PMC6246677

[B31] Shin H.-C. Roth H. R. Gao M. Lu L. Xu Z. Nogues I. (2016). Deep convolutional neural networks for computer-aided detection: CNN architectures, dataset characteristics and transfer learning. *IEEE Trans. Med. Imaging* 35 1285–1298. 10.1109/TMI.2016.2528162 26886976 PMC4890616

[B32] Simonyan K. Vedaldi A. Zisserman A. (2013). Deep inside convolutional networks: visualising image classification models and saliency maps. *arXiv [Preprint].*

[B33] Skarpaas T. L. Jarosiewicz B. Morrell M. J. (2019). Brain-responsive neurostimulation for epilepsy (RNS§System). *Epilepsy Res.* 153 68–70. 10.1016/j.eplepsyres.2019.02.003 30850259

[B34] Snell J. Swersky K. Zemel R. (2017). Prototypical networks for few-shot learning. *Adv. Neural Inf. Process. Syst. arXiv*. arXiv:1703.05175

[B35] Sun F. T. Arcot Desai S. Tcheng T. K. Morrell M. J. (2018). Changes in the electrocorticogram after implantation of intracranial electrodes in humans: the implant effect. *Clin. Neurophysiol.* 129 676–686. 10.1016/j.clinph.2017.10.036 29233473

[B36] Thodoroff P. Pineau J. Lim A. (2016). “Learning robust features using deep learning for automatic seizure detection,” in *Paper Presented at the Machine Learning for Healthcare Conference.* Los Angeles, CA.

[B37] Tsinalis O. Matthews P. M. Guo Y. (2016). Automatic sleep stage scoring using time-frequency analysis and stacked sparse autoencoders. *Ann. Biomed. Eng.* 44 1587–1597. 10.1007/s10439-015-1444-y 26464268 PMC4837220

[B38] Ullah I. Hussain M. Aboalsamh H. (2018). An automated system for epilepsy detection using EEG brain signals based on deep learning approach. *Exp. Syst. Appl.* 107 61–71. 10.1016/j.eswa.2018.04.021

[B39] Ung H. Baldassano S. N. Bink H. Krieger A. M. Williams S. Vitale F. (2017). Intracranial EEG fluctuates over months after implanting electrodes in human brain. *J. Neural Eng.* 14:056011. 10.1088/1741-2552/aa7f40 28862995 PMC5860812

[B40] Verleysen M. François D. (2005). “The curse of dimensionality in data mining and time series prediction,” in *Proceedings of the International Work-Conference on Artificial Neural Networks*, (Berlin: Springer). 10.1007/11494669_93

[B41] Vinyals O. Blundell C. Lillicrap T. Kavukcuoglu K. Wierstra D. (2016). Matching networks for one shot learning. *Adv. Neural Inf. Process. Syst. arxiv*. arXiv:1606.04080

[B42] Vrbancic G. Podgorelec V. (2018). Automatic classification of motor impairment neural disorders from EEG signals using deep convolutional neural networks. *Elektronika ir Elektrotechnika* 24 3–7. 10.5755/j01.eie.24.4.21469

[B43] Wang J. Song Y. Leung T. Rosenberg C. Wang J. Philbin J. (2014). “Learning fine-grained image similarity with deep ranking,” in *Proceedings of the IEEE Conference on Computer Vision and Pattern Recognition*, (New York, NY: IEEE). 10.1109/CVPR.2014.180

[B44] Yildirim Ö Baloglu U. B. Acharya U. R. (2020). A deep convolutional neural network model for automated identification of abnormal EEG signals. *Neural Comput. Appl.* 32 15857–15868. 10.1007/s00521-018-3889-z

[B45] Zeng H. Yang C. Dai G. Qin F. Zhang J. Kong W. (2018). EEG classification of driver mental states by deep learning. *Cogn. Neurodyn.* 12 597–606. 10.1007/s11571-018-9496-y 30483367 PMC6233328

