# Supplementary material for: A High Accuracy Electrographic Seizure Classifier Trained Using Semi-Supervised Labeling Applied to a Large Spectrogram Dataset
Source: Front Neurosci. 2021 Jun 28;15:667373. doi: 10.3389/fnins.2021.667373 (PMC8273175; doi:10.3389/fnins.2021.667373)
Supplement: Supplementary file 1 [file Data_Sheet_1.docx]

**Supplementary Data**

**Supplementary Figures**

**
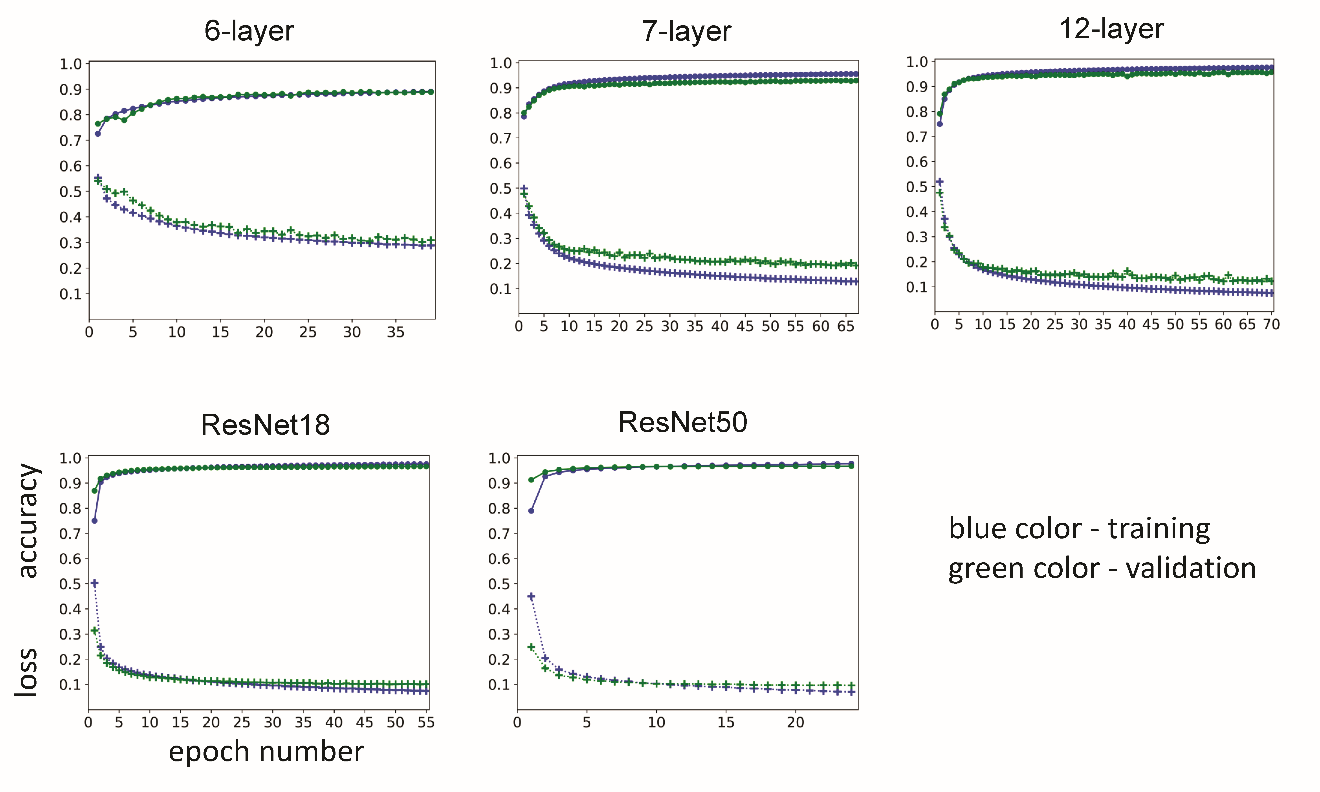
**

**Supplementary Figure 1.** Example training and validation curves and losses over training
epochs for the different types of models used in this paper.


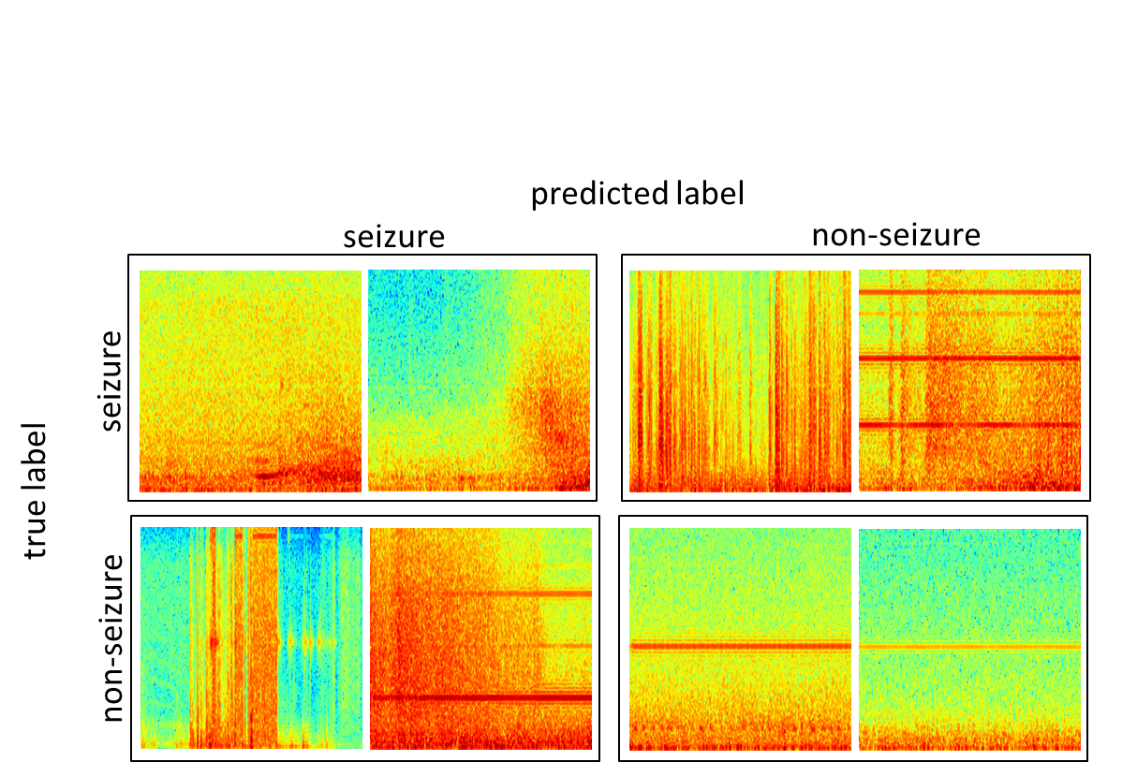


**Supplementary Figure 2.** Example correctly classified and misclassified EEG signals from the TUH EEG Seizure Corpus dataset.

**Supplementary Tables**

| **Supplementary Table 1.** ECoG channel-level and record-level test accuracies and F_1_ scores for models trained with the Adam optimizer. | | | | | | | | | |
| --- | --- | --- | --- | --- | --- | --- | --- | --- | --- |
| **Model** | **Run** | **Test accuracy% on  held-out  ECoG channels** | | | **F_1_ score** | **Test accuracy% on  independent expert labeled  ECoG records** | | | **F_1_ score** |
|  |  | **Overall** | **NSZ  Class** | **SZ  Class** |  | **Overall** | **NSZ  Class** | **SZ  Class** |  |
| **6-layer**  LR: 10^-6^  Opt: Adam | 1 | 91.09 | 94.75 | 87.43 | 90.38 | 89.49 | 93.09 | 85.88 | 83.70 |
|  | 2 | 88.93 | 94.95 | 82.91 | 85.61 | 86.50 | 93.99 | 79.01 | 84.66 |
|  | 3 | 76.27 | 89.90 | 62.64 | 67.70 | 75.90 | 79.28 | 72.52 | 72.94 |
|  | 4 | 91.99 | 93.97 | 90.02 | 87.28 | 87.72 | 99.10 | 76.34 | 86.02 |
|  | 5 | 88.21 | 92.92 | 83.50 | 85.15 | 88.17 | 95.80 | 80.53 | 86.65 |
|  | **Ave** | **87.30** | **93.29** | **81.30** | **83.23** | **85.55** | **92.25** | **78.85** | **83.70** |
| **7-layer**  LR: 10^-6^  Opt: Adam | 1 | 92.94 | 95.42 | 90.46 | 92.43 | 92.44 | 95.20 | 89.69 | 91.62 |
|  | 2 | 92.15 | 95.91 | 88.40 | 89.69 | 90.33 | 92.49 | 88.17 | 89.19 |
|  | 3 | 88.71 | 97.02 | 80.39 | 85.99 | 87.46 | 98.20 | 76.72 | 85.71 |
|  | 4 | 93.80 | 95.70 | 91.90 | 90.32 | 92.55 | 95.80 | 89.31 | 91.76 |
|  | 5 | 91.51 | 91.95 | 91.08 | 88.68 | 91.93 | 93.39 | 90.46 | 90.98 |
|  | **Ave** | **91.82** | **95.20** | **88.45** | **89.42** | **90.94** | **95.02** | **86.87** | **89.85** |
| **12-layer**  LR: 10^-6^  Opt: Adam | 1 | 95.45 | 97.39 | 93.51 | 95.17 | 94.23 | 96.10 | 92.37 | 93.62 |
|  | 2 | 94.75 | 95.78 | 93.73 | 92.47 | 92.95 | 90.09 | 95.80 | 91.94 |
|  | 3 | 92.14 | 97.41 | 86.87 | 90.20 | 89.68 | 97.30 | 82.06 | 88.48 |
|  | 4 | 95.20 | 96.62 | 93.78 | 92.45 | 95.60 | 97.30 | 93.89 | 95.16 |
|  | 5 | 94.73 | 96.85 | 92.60 | 93.44 | 93.90 | 94.29 | 93.51 | 93.16 |
|  | **Ave** | **94.45** | **96.81** | **92.10** | **92.74** | **93.27** | **95.02** | **91.53** | **92.47** |
| **RN18**  LR: 10^-7^  Opt: Adam | 1 | 95.97 | 97.01 | 94.92 | 95.71 | 94.69 | 91.29 | 98.09 | 93.80 |
|  | 2 | 95.29 | 96.35 | 94.24 | 93.30 | 92.25 | 86.79 | 97.71 | 91.10 |
|  | 3 | 95.29 | 97.83 | 92.75 | 93.89 | 93.45 | 89.19 | 97.71 | 92.42 |
|  | 4 | 94.96 | 95.52 | 94.39 | 91.43 | 96.04 | 93.99 | 98.09 | 95.36 |
|  | 5 | 95.93 | 97.83 | 94.03 | 95.03 | 95.33 | 92.19 | 98.47 | 94.51 |
|  | **Ave** | **95.49** | **96.91** | **94.06** | **93.87** | **94.35** | **90.69** | **98.02** | **93.44** |
| **RN50**  LR: 10^-7^  Opt: Adam | 1 | 95.93 | 96.63 | 95.23 | 95.66 | 92.36 | 87.39 | 97.33 | 91.23 |
|  | 2 | 95.66 | 96.13 | 95.19 | 93.58 | 92.36 | 87.39 | 97.33 | 91.23 |
|  | 3 | 95.10 | 98.13 | 92.07 | 93.84 | 89.48 | 80.48 | 98.47 | 88.21 |
|  | 4 | 95.44 | 96.07 | 94.82 | 92.32 | 94.07 | 93.09 | 95.04 | 93.26 |
|  | 5 | 95.97 | 97.66 | 94.28 | 95.02 | 94.94 | 93.69 | 96.18 | 94.21 |
|  | **Ave** | **95.62** | **96.92** | **94.32** | **94.08** | **92.64** | **88.41** | **96.87** | **91.63** |
| ECoG = electrocorticographic; NSZ = non-seizure; SZ = seizure. | | | | | | | | | |
|  | | | | | | | | | |

| **Supplementary Table 2.** Number of seizure and non-seizure ECoG channels used for training and validation in every fold for 10-80 patient splits used in training. | | | | | | |
| --- | --- | --- | --- | --- | --- | --- |
| **Fold** | **# of training patients** | **# of SZ ECoG  channels used  for training** | **# of NSZ ECoG  channels available  for training*** | **# of  validation patients** | **# of SZ ECoG  channels used  for validation** | **# of NSZ ECoG  channels used  for validation** |
| 1 | 10 | 9,901 | 53,905 | 5 | 6,010 | 8,266 |
| 2 | 10 | 14,343 | 12,215 | 5 | 4,737 | 2,864 |
| 3 | 10 | 14,818 | 18,202 | 5 | 6,030 | 12,762 |
| 4 | 10 | 15,180 | 19,876 | 5 | 4,669 | 7,082 |
| 5 | 10 | 18,103 | 12,117 | 5 | 4,699 | 7,610 |
| **Avg** | **10** | **14,469** | **23,263** | **5** | **5,229** | **7,717** |
| 1 | 20 | 20,586 | 63,310 | 5 | 10,153 | 5,341 |
| 2 | 20 | 27,411 | 27,251 | 5 | 6,527 | 45,748 |
| 3 | 20 | 26,025 | 52,849 | 5 | 4,817 | 3,242 |
| 4 | 20 | 26,488 | 85,477 | 5 | 8,285 | 2,865 |
| 5 | 20 | 29,812 | 59,587 | 5 | 5,403 | 21,308 |
| **Avg** | **20** | **26,064** | **57,695** | **5** | **7,037** | **15,701** |
| 1 | 30 | 35,629 | 86,134 | 6 | 9,780 | 37,154 |
| 2 | 30 | 41,278 | 102,228 | 6 | 10,993 | 7,708 |
| 3 | 30 | 35,157 | 64,492 | 6 | 7,994 | 31,405 |
| 4 | 30 | 44,365 | 93,134 | 6 | 7,090 | 16,690 |
| 5 | 30 | 40,351 | 90,301 | 6 | 6,348 | 4,561 |
| **Avg** | **30** | **39,356** | **87,258** | **6** | **8,441** | **19,504** |
| 1 | 40 | 46,069 | 114,424 | 8 | 11,572 | 8,077 |
| 2 | 40 | 56,006 | 137,382 | 8 | 8,701 | 15,349 |
| 3 | 40 | 46,857 | 93,823 | 8 | 16,187 | 25,777 |
| 4 | 40 | 58,837 | 116,185 | 8 | 6,022 | 8,993 |
| 5 | 40 | 50,883 | 104,498 | 8 | 11,881 | 7,866 |
| **Avg** | **40** | **51,730** | **113,262** | **8** | **10,873** | **13,212** |
| 1 | 50 | 59,858 | 122,049 | 10 | 21,900 | 28,266 |
| 2 | 50 | 72,548 | 153,074 | 10 | 10,291 | 20,880 |
| 3 | 50 | 69,077 | 133,020 | 10 | 14,689 | 13,900 |
| 4 | 50 | 68,794 | 130,505 | 10 | 10,049 | 15,590 |
| 5 | 50 | 64,892 | 130,313 | 10 | 11,738 | 49,796 |
| **Avg** | **50** | **67,034** | **133,792** | **10** | **13,733** | **25,686** |
| 1 | 60 | 81,758 | 150,315 | 12 | 13,660 | 17,373 |
| 2 | 60 | 82,839 | 173,954 | 12 | 21,232 | 29,806 |
| 3 | 60 | 83,766 | 146,920 | 12 | 16,293 | 27,283 |
| 4 | 60 | 78,843 | 146,095 | 12 | 14,079 | 37,315 |
| 5 | 60 | 76,630 | 180,109 | 12 | 11,497 | 16,212 |
| **Avg** | **60** | **80,767** | **159,479** | **12** | **15,352** | **25,598** |
| 1 | 70 | 93,512 | 166,765 | 14 | 19,487 | 30,642 |
| 2 | 70 | 102,918 | 203,527 | 14 | 15,837 | 32,143 |
| 3 | 70 | 99,096 | 165,567 | 14 | 15,197 | 70,445 |
| 4 | 70 | 90,947 | 181,013 | 14 | 22,468 | 42,104 |
| 5 | 70 | 87,370 | 192,513 | 14 | 11,701 | 19,878 |
| **Avg** | **70** | **94,769** | **181,877** | **14** | **16,938** | **39,042** |
| 1 | 80 | 107,645 | 185,898 | 16 | 20,650 | 49,532 |
| 2 | 80 | 113,617 | 233,113 | 16 | 18,398 | 27,739 |
| 3 | 80 | 111,101 | 232,594 | 16 | 15,110 | 33,057 |
| 4 | 80 | 111,422 | 209,590 | 16 | 18,677 | 34,821 |
| 5 | 80 | 97,600 | 211,453 | 16 | 30,445 | 42,039 |
| **Avg** | **80** | **108,277** | **214,530** | **16** | **20,656** | **37,438** |
| Avg = average; ECoG = electrocorticographic; NSZ = non-seizure; SZ = seizure.  * Note that the number of training examples in the non-seizure classification (i.e., the majority class) was always downsampled to match the number of examples in the seizure classification. | | | | | | |
|  | | | | | | |

| **Table 3A. Class-balanced accuracy percentage (mean ± standard deviation) as a function of the number of patients’ data used for training.** | | | | | | | | |
| --- | --- | --- | --- | --- | --- | --- | --- | --- |
| **Model** | **Number of patients’ data used for training** mean (standard deviation) | | | | | | | |
|  | **10** | **20** | **30** | **40** | **50** | **60** | **70** | **80** |
| 6 layer CNN | 73.42  (5.2) | 75.75  (3.0) | 78.97  (6.0) | 80.44  (4.4) | 80.5  (5.0) | 83.58  (5.9) | 86.24  (2.7) | 85.85  (1.1) |
| 7 layer CNN | 75.08  (5.8) | 82.63  (2.8) | 85.26  (4.5) | 84.63  (6.6) | 90.4  (1.6) | 90.68  (1.7) | 90.69  (1.2) | 89.85  (1.7) |
| 12 layer CNN | 76.14  (6.5) | 84.93  (2.4) | 85.98  (3.1) | 89.88  (1.6) | 91.93  (1.8) | 92.24  (2.2) | 93.44  (0.5) | 93.59  (0.8) |
| ResNet18 | 85.95  (5.1) | 90  (1.9) | 90.46  (1.3) | 91.34  (0.7) | 91.28  (0.7) | 92.41  (1.3) | 92.71  (0.8) | 93.73  (0.8) |
| ResNet50 | 88.41  (2.3) | 89.86  (2.0) | 91.35  (1.6) | 91.33  (0.7) | 92.55  (1.5) | 93.08  (1.1) | 93.37  (1.0) | 93.69  (0.6) |
| CNN = convolutional neural networks. | | | | | | | | |
|  | | | | | | | | |

| **Table 3B. Number of training epochs (mean ± standard deviation)** | | | | | | | | |
| --- | --- | --- | --- | --- | --- | --- | --- | --- |
| **Model** | **Number of patients’ data used for training** mean (standard deviation) | | | | | | | |
|  | **10** | **20** | **30** | **40** | **50** | **60** | **70** | **80** |
| 6 layer CNN | 35.2  (26.5) | 26.0  (30.8) | 34.0  (25.0) | 46.0  (25.5) | 31.8  (26.7) | 41.0  (27.0) | 58.0  (10.4) | 35.8  (14.3) |
| 7 layer CNN | 27.2  (26.8) | 30.5  (17.5) | 51.0  (21.9) | 46.7  (27.8) | 53.3  (12.2) | 54.0  (14.8) | 48.5  (17.8) | 47.0  (25.9) |
| 12 layer CNN | 31.2  (26.8) | 34.7  (23.8) | 37.2  (18.9) | 49.7  (16.5) | 40.2  (17.4) | 31.0  (8.6) | 33.3  (8.8) | 29.0  (12.4) |
| ResNet18 | 50.3  (26.3) | 34.3  (20.8) | 26.3  (11.6) | 52.0  (13.5) | 36.7  (18.3) | 42.5  (15.4) | 37.8  (8.2) | 31.4  (9.7) |
| ResNet50 | 50.5  (9.2) | 29.3  (13.8) | 20.5  (10.1) | 34.7  (4.8) | 21.5  (10.6) | 24.0  (3.9) | 23.7  (2.1) | 16.2  (4.9) |
| CNN = convolutional neural networks. | | | | | | | | |
|  | | | | | | | | |

Python code lines (tensorflow graph) for generating 3-color channel spectrograms for unsupervised clustering of ECoG records.

#sig is the time series waveform

import tensorflow

sig = tensorflow.placeholder(tensorflow.float32, [1, None])

spectrogram = tensorflow.contrib.signal.stft(sig, 256, 128)

mag = tensorflow.abs(spectrogram)

#expand then resize then gray

channel = tensorflow.expand_dims(mag, axis=3)

resize = tensorflow.image.resize_nearest_neighbor(channel, [299, 299])

rgb = tensorflow.image.grayscale_to_rgb(resize)

Python code lines for generating and saving RGB images of spectrograms for training and testing electrographic seizure classifiers.

#x is the time series waveform

#fig_name = path to save file + filename

from scipy import signal as sg

import matplotlib.pyplot as plt

import numpy as np

fs = 250

width = 2.99

height = 2.99

f, t, Sxx = sg.spectrogram(x, fs, nfft = 512, noverlap=128*0.80, nperseg = 128)

fig, ax = plt.subplots(figsize=(width, height))

ax = fig.add_axes([0, 0, 1, 1])

ax.axis('off')

ax.set_ylim(0, 120)

Sxx = 10 * np.log10(Sxx + 0.000001)

ax.pcolormesh(t, f, Sxx, cmap = 'jet')

fig.savefig(fig_name, dpi = fig.dpi)

plt.close('all')
